# The IKKα-regulated microRNA miR-9-5p mediates lung cancer growth and invasiveness via CDH1/Wnt/β-catenin signalling

**DOI:** 10.1038/s41420-026-03195-8

**Published:** 2026-06-17

**Authors:** Simoni Besta, Eugenia Roupakia, Zoi Kanaki, Giannis Vatsellas, Mark Samuels, Spyros Foutadakis, Maria Tokamani, Alessandro Barbon, Raphael Sandaltzopoulos, Dimitris C. Kanellis, Jiri Bartek, Maria Hatziapostolou, Christos Polytarchou, Dimitris Thanos, Apostolos Klinakis, Evangelos Kolettas

**Affiliations:** 1 Laboratory of Biology, School of Medicine, Faculty of Health Sciences, and Institute of Biosciences, Centre for Research and Innovation, University of Ioannina, University Campus, Ioannina, Greece; 2 Biomedical Research Institute, Foundation for Research and Technology, University of Ioannina Campus, Ioannina, Greece; 3 Centre of Basic Research, Biomedical Research Foundation, Academy of Athens, Athens, Greece; 4 Greek Genome Center, Biomedical Research Foundation, Academy of Athens, Athens, Greece; 5 Department of Biochemistry and Biomedicine, School of Life Sciences, University of Sussex, Brighton, UK; 6 4th Department of Internal Medicine, School of Medicine, National and Kapodistrian University of Athens, Athens, Greece; 7 Department of Molecular Biology and Genetics, Democritus University of Thrace, Alexandroupolis, Thrace Greece; 8 Department of Molecular and Translational Medicine, University of Brescia, Brescia, Italy; 9 Science for Life Laboratory, Division of Genome Biology, Department of Medical Biochemistry and Biophysics, Karolinska Institutet, Stockholm, Sweden; 10 Genome Integrity, Danish Cancer Institute, Danish Cancer Society, Copenhagen, Denmark; 11 Department of Biosciences, John van Geest Cancer Research Centre, Centre for Systems Health and Integrated Metabolic Research, School of Science and Technology, Nottingham Trent University, Nottingham, UK

**Keywords:** Cell invasion, Non-small-cell lung cancer, Cell growth, Cell signalling

## Abstract

IKKα is a serine/threonine kinase that acts as a tumour suppressor in non-small cell lung cancer (NSCLC) in an NF-κΒ-independent manner, however, little is known about its downstream signalling pathways. To interrogate the IKKα signalling network in NSCLC, we investigated the impact of IKKα silencing on the miRNA expression profile of NSCLC cells. Nanostring miRNome analysis identified miR-9-5p, a known oncomir, as the top upregulated miRNA upon IKKα depletion. We show that overexpression of miR-9-5p in human lung cancer cells increases cell migration and invasion and promotes epithelial-to-mesenchymal cell transition (EMT) through loss of E-cadherin and activation of the Akt1/β-catenin pathway. In vivo tumour xenograft models show that overexpression of miR-9-5p promotes tumour growth and widespread transcriptomic reprogramming, altering the expression of genes related to EMT, apoptosis and NF-κB signalling. Overall, we show that miR-9-5p acts as an oncogenic effector upon IKKα loss, promoting tumour progression through EMT and Akt-1/GSK-3β/β-catenin pathway activation, leading to increased invasiveness and tumour growth.

## Introduction

Lung cancer is the leading cause of cancer death and the most common cancer type worldwide [1]. Non-small cell lung cancer (NSCLC) is the most common lung cancer type, and is characterised by different histopathological subtypes, with lung adenocarcinoma (LUAD) and lung squamous cell carcinoma (LUSC) being the major subtypes. Despite recent advances in targeted therapies, survival rates for the disease remain low. Understanding the signalling pathways driving lung cancer is therefore essential for the development of novel therapies.

The nuclear factor kappa B (NF-κB) signalling pathways and upstream activating Ser/Thr kinases, IκB Kinase α (IKKα) and IκB Kinase β (IKKβ) play key roles in lung cancer onset, development and progression by regulating the expression of target genes. In cancer, NF-κB transcription factors (TFs) and their upstream signalling components exhibit either tumour-promoting or growth-suppressive activities depending on the cellular context [2–4]. NF-κB TFs are part of a regulatory network, and NF-κB responses are typically self-limiting through the induction of negative feedback loops; however, these auto-regulatory loops often become deregulated in cancer [3, 5], contributing to lung tumour initiation, development and progression [6–12].

IKKα activates non-canonical NF-κB signalling, but also has several important NF-κB-independent functions in signal transduction, gene expression, cell cycle regulation and cell death, impacting normal physiology and tumour development [13**–**16]. These diverse mechanisms suggest that IKKα may regulate oncogenesis through multiple downstream mechanisms. In the nucleus, IKKα regulates the expression of target genes by phosphorylating histone H3, the transcriptional co-repressor silencing mediator of retinoic acid and thyroid hormone receptor (SMRT) and the coactivator CREB-binding protein (CBP), inducing the expression of both NF-κB-dependent and -independent genes [2, 14, 17]. Additionally, most functional studies show that IKKα is a tumour suppressor in both LUAD [8, 18, 19] and LUSC [4, 20]. As such, IKKα is frequently downregulated in lung cancers compared to normal tissue and undergoes hemizygous deletion in approximately 22% of LUAD cases [18, 20]. IKKα loss promotes *Kras*-initiated NSCLC development through a redox regulatory pathway. This involves upregulation of NOX2 and downregulation of NRF2, leading to ROS accumulation and acceleration of NSCLC [18]. We previously demonstrated that IKKα silencing causes upregulation of HIF-1α and hypoxia-mediated processes, supporting NSCLC growth during hypoxic conditions in vivo in an NF-κΒ-independent manner [8]. However, beyond this, the downstream pathways of IKKα in cancer remain poorly understood. As microRNAs (miRNAs) are increasingly recognised as key players in cancer [21], we explored how IKKα loss may impact the miRNA expression profile of lung cancer cells.

By binding to cis-regulatory mRNA elements usually found in the 3’ untranslated region, miRNAs work at the post-transcriptional level, negatively regulating mRNA translation and stability [21, 22]. Interestingly, the interplay between TFs and miRNAs is a common regulatory mechanism in many biological processes, including in NF-κB signalling where it regulates cell proliferation, survival, stress, inflammation, senescence and tumour progression [5, 23, 24]. IKKα is also negatively regulated by miR-15a, miR-16 and miR-223 which bind to the 3′ UTR of the IKKα mRNA, leading to changes in the expression of non-canonical NF-κB target genes during macrophage differentiation [24, 25].

As IKKα is frequently downregulated in lung cancer and accumulating evidence suggests that this event can promote oncogenesis independently of NF-κB signalling, in this study we focused on the effects of IKKα depletion on miRNA expression and their mechanisms of action, rather than the pathway as a whole. To further explore the tumour suppressor functions of IKKα in NSCLC, we investigated the miRNA expression profile of IKKα-deficient lung cancer cells, revealing miR-9-5p as an IKKα-regulated, tumour-promoting miRNA. Furthermore, overexpression of miR-9-5p increases cell migration, invasion and tumour growth, accompanied by modulation of EMT markers and activation of Akt/GSK-3β/β-catenin signalling. In addition, we show that miR-9-5p upregulation produces profound changes on the transcriptome of lung cancer cells, altering the expression of genes involved in diverse cellular processes, including apoptosis, EMT and NF-κB signalling [5]. Overall, we show that IKKα loss drives miR-9-5p upregulation, supporting cancer progression through widespread transcriptomic changes and EMT.

## Results

### Expression profiling of IKKα-regulated miRNAs

**Fig. 1:**
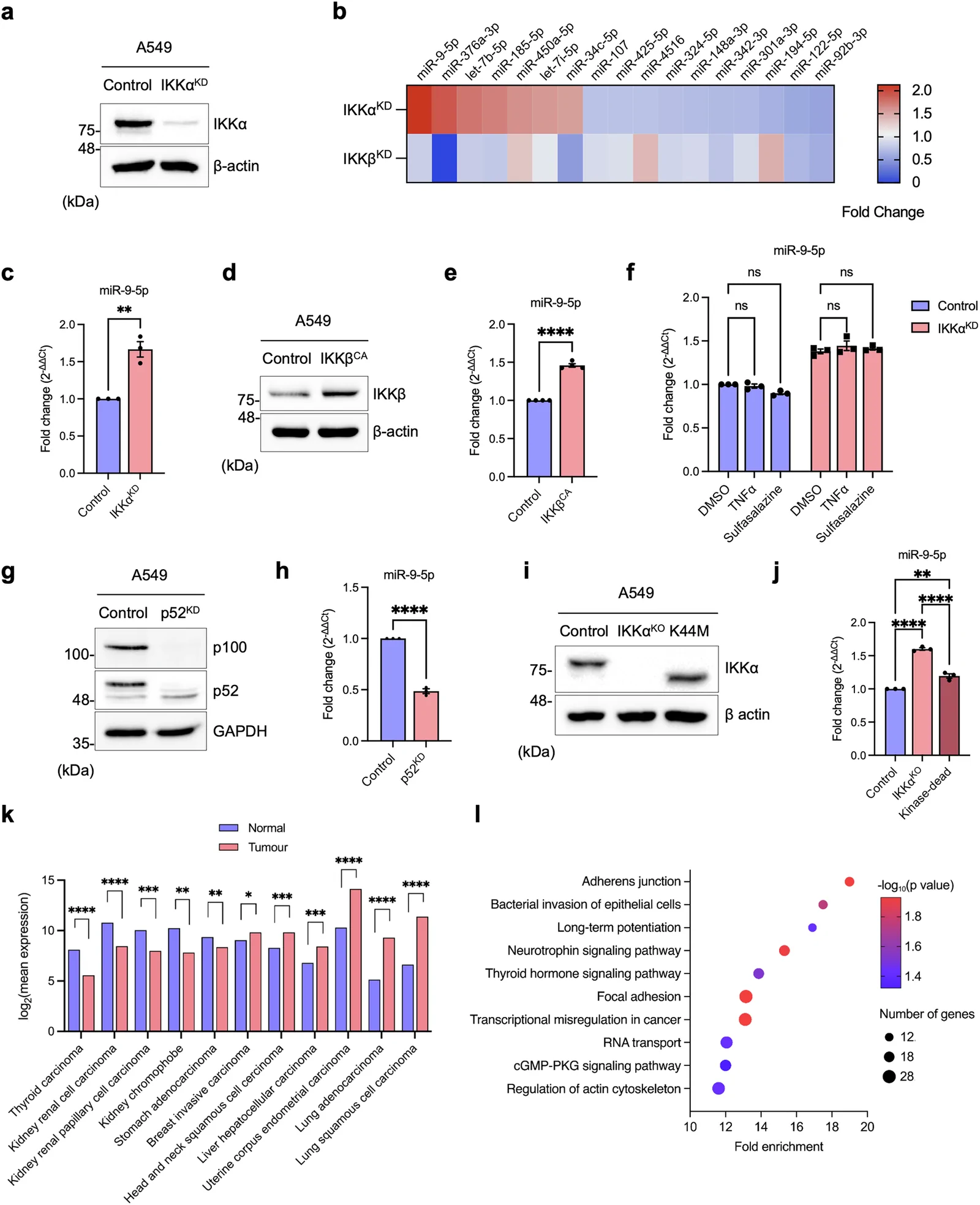
Nanostring miRNome analysis of A549 IKKα knockdown cell lines. **a** Western blot for the expression of IKKα in A549 control and IKKα^KD^ cells. β-actin was used as a loading control. **b** Heatmap of miRNA expression levels in A549 IKKα^KD^ and IKKβ^KD^ cell lines. This figure shows selected miRNAs with a fold change >1.5 or <0.75. **c** RT-qPCR analysis of miR-9-5p expression levels in A549 IKKα^KD^ compared to control cells. Graphs show mean ± SEM, **p < 0.01 (two-tailed unpaired Student’s t test), *n* = 3 **d** Western blot for IKKβ in control and IKKβ-overexpressing cells (IKKβ^CA^). β-actin was used as a loading control. **e** RT-qPCR analysis of miR-9-5p **e**xpression levels in A549 control and IKKβ^CA^ cells. Graphs show mean ± SEM, ****p < 0.0001 (two-tailed unpaired Student’s t test), *n* = 3. **f** RT-qPCR analysis of A549 control and IKKα^KD^ cells treated with either DMSO, 10 ng/ml TNFα, or 2 μM Sulfasalazine. Graphs show mean ± SEM, ns, non-significant (two-way ANOVA), *n* = 3. **g** Western blot for p52/p100 in control and p52^KD^ cells. GAPDH was used as a loading control. **h** RT-qPCR analysis of miR-9-5p levels in control and p52^KD^ cells. Graphs show mean ± SEM, ****p < 0.0001 (two-tailed unpaired Student’s t test) *n* = 3. **i** Western blot for IKKα in control, IKKα knockout (IKKα^KO^) and IKKα^KO^ cells expressing a kinase-dead mutant IKKα (K44M). β-actin was used as a loading control. **j** RT-qPCR analysis of miR-9-5p expression levels in A549 control, IKKα knockout and IKKα kinase-dead cells. Graphs show mean ± SEM, **p < 0.01, ****p < 0.0001 (two-way ANOVA), *n* = 3. **k** Mean expression of miR-9-5p in tumours compared to normal adjacent tissue, based on TCGA data. Analysis was performed by OncoMir [37]. Student’s t test (*p < 0.05, **p < 0.01, ***p < 0.001, ****p < 0.0001). **l** KEGG pathway analysis of top miR-9-5p targets. Analysis was performed by miRPath v4.0 using experimentally supported target genes from TarBase v8.0 [30, 38]. FDR-adjusted p-values are shown.

Previous studies from our laboratory have shown that IKKα acts as an important NSCLC tumour suppressor by regulating hypoxia-mediated processes, enhancing tumour growth in vivo [8]. To further investigate the impact of IKKα on lung cancer development, we generated A549 ΙΚΚα knockdown (ΙΚΚα^KD^) and empty vector control cell lines to study the impact of ΙΚΚα-dependent alterations on the miRNA expression profiles (Fig. 1a). Total RNA was isolated from cells, enriched for miRNAs and then subjected to miRNome profiling using digital molecular barcoding-based Nanostring technology. Data were analysed using the nSolver software [26–28]. In IKKα^KD^ cells, 125 of the panel of 800 miRNAs were expressed at levels above the detection threshold (Table S1). In IKKα^KD^ cells, miR-92b-3p and miR-122-5p exhibited the strongest downregulation, while miR-9-5p and miR-376a-3p exhibited the strongest upregulation compared to control cells. A549 IKKβ^KD^ cells (Supplementary Fig. 1a) were analysed in parallel and compared to IKKα^KD^ cells (Fig. 1b, and Table S1). The levels of miR-9-5p were reduced in the IKKβ^KD^ cells, compared to control cells, suggesting that IKKβ plays a different role to IKKα in the regulation of miR-9-5p, generating an opposite effect. The common experimentally supported mRNA targets of the differentially expressed miRNAs in the A549 IKKα^KD^ cells were identified using miRNet and TarBase (v9.0) [29, 30] (Supplementary Fig. 1b). Pathway analysis showed enrichment in p53 signalling, FoxO signalling, cell cycle, adherens junctions and cell senescence pathways (Supplementary Fig. 1c). As the top hit, miR-9-5p was selected for further investigation as it has been previously implicated in lung cancer malignancy [31–34] and RT-qPCR validated the upregulation of miR-9-5p in A549 IKKα^KD^ cells (Fig. 1c).

To further explore the interplay between NF-κB factors and miR-9-5p expression levels, we generated A549 cells overexpressing a constitutively active form of IKKβ (A549 IKKβ^CA^) (Fig. 1d). This resulted in an increase in miR-9-5p expression compared to control cells, as expected based on the miRNome profiling (Fig. 1e). In addition, A549 control and IKKα^KD^ cells were treated with either TNFα, an inducer of the canonical NF-κB pathway, or sulfasalazine, an inhibitor of IKK/NF-κB signalling [35, 36] (Supplementary Fig. 1d). Neither treatment altered the levels of miR-9-5p in either control or IKKα^KD^ cells, suggesting that, in contrast to chronic canonical NF-κB activation by overexpression of IKKβ^CA^, acute activation or inhibition of the pathway is not sufficient to affect miR-9-5p levels in this system (Fig. 1f). As IKKα is the key regulator of non-canonical NF-κB signalling, we also assessed whether p52 contributes to miR-9-5p regulation. Silencing of p100/p52 in A549 cells caused a reduction in miR-9-5p levels, indicating that p52 positively regulates miR-9-5p expression (Fig. 1g, h). This opposite effect to the increase observed upon IKKα silencing suggests that these factors may regulate miR-9-5p through distinct mechanisms, and that the effect is NF-κB-independent. Finally, we examined whether the kinase activity of IKKα is required for the regulation of miR-9-5p. Using a CRISPR-generated A549 IKKα^KO^ cell line, we re-expressed a kinase-dead mutant of IKKα (IKKα^K44M^) and measured the levels of miR-9-5p (Fig. 1i, j). Re-expression of IKKα^K44M^ partially rescued the expression of miR-9-5p, suggesting that the regulation of miR-9-5p is at least partly dependent by the kinase activity, although incomplete rescue indicates that additional mechanisms may contribute to IKKα-mediated regulation. Together, these results indicate that IKKα negatively regulates miR-9-5p, whereas other NF-κB signalling components can also impact miR-9-5p levels in lung cancer cells.

Next, the OncoMiR database [37] was used to assess the expression of miR-9-5p in different forms of cancer using the Cancer Genome Atlas (TCGA) datasets (Fig. 1k). Interestingly, LUAD and LUSC showed the highest increase in miR-9-5p expression in tumour samples compared to normal tissue among the different cancer types analysed, suggesting a key role in lung cancer development. To explore the biological processes associated with miR-9-5p experimentally supported targets, KEGG pathway analysis was performed using miRPath [38], highlighting adherens junction, focal adhesion and transcriptional misregulation in cancer as among the top enriched terms (Fig. 1l). Taken together, these findings suggest that miR-9-5p upregulation is functionally linked to several oncogenic processes and may have an important role in lung cancer progression.

### Upregulation of miR-9-5p promotes invasiveness through the regulation of EMT

**Fig. 2:**
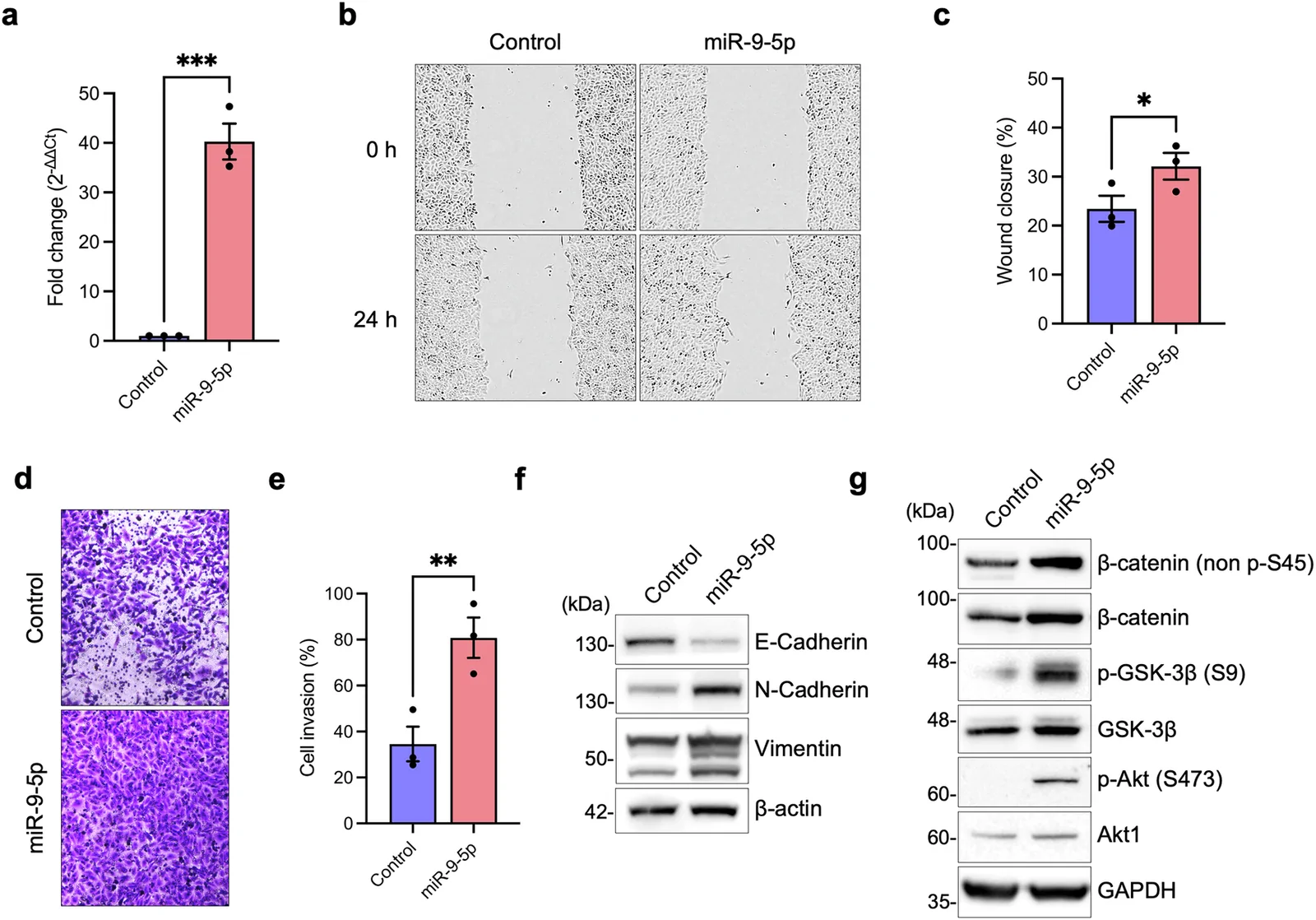
Overexpression of miR-9-5p promotes cell invasion and migration and alterations in EMT markers. **a** RT-qPCR analysis of A549 stable cell lines overexpressing miR-9-5p. Graphs show mean ± SEM. ***p < 0.001 (two-tailed unpaired Student’s t test), *n* = 3. **b** Cell migration scratch assay of A549 control and miR-9-5p-overexpressing cell lines. Representative images are shown, taken at 0 h and 24 h after the scratch. **c** Quantification of the scratch assays. Graphs show mean ± SEM, *p < 0.05 (two-tailed unpaired Student’s t test), n = 3 biological repeats. **d** Matrigel invasion assay showing increase in invasiveness with miR-9-5p overexpression. Representative images are shown. **e** Quantification of invasion assays, **p < 0.01 (two-tailed unpaired Student’s t test), *n* = 3 independent experiments. **f** Western blot of EMT markers E-cadherin, N-cadherin and vimentin in A549 control and miR-9-5p overexpressing cell lines. β-actin was used as a loading control. **g** Western blot of proteins related to Wnt signalling in A549 control and miR-9-5p overexpressing cell lines. GAPDH was used as a loading control.

To further explore the role of miR-9-5p in lung cancer, we established a miR-9-5p-overexpressing clonal A549 cell line using Twl-PGKgfp-H1 (control) and Twl-PGKgfp-H1-miR-9-5p (miR-9-5p) lentivectors (Fig. 2a) (Supplementary Fig. 2a) [39]. Our pathway analysis of the top experimentally supported target genes of miR-9-5p showed an enrichment in genes involved in focal adhesion and adherens junctions (Fig. 1f), suggesting alterations in EMT-related processes. To assess this, cell migration and invasion assays were performed, showing that overexpression of miR-9-5p increased migration in scratch assays (Fig. 2b, c) and promoted cell invasion in transwell-based invasion assays (Fig. 2d, e). Immunoblotting showed downregulation of the epithelial cell marker E-cadherin and upregulation of the mesenchymal cell markers N-cadherin and vimentin upon miR-9-5p overexpression (Fig. 2f). E-cadherin is typically bound to β-catenin near the cell membrane, where it forms cadherin cell junctions [40–42]. Overexpression of miR-9-5p resulted in increased levels of active (non-phospho-Ser45) and total β-catenin (Fig. 2g), indicating enhanced β-catenin stability and suggesting activation of the canonical Wnt signalling pathway.

Furthermore, we found that levels of inactive phosphorylated (Ser9) glycogen synthase kinase-3β (GSK-3β) were increased with miR-9-5p overexpression, while total GSK-3β remained unchanged, suggesting decreased inhibition of β-catenin. As Akt1 is the primary kinase phosphorylating GSK-3β, we examined total and phosphorylated Akt1 (Ser473) and observed significantly elevated active Akt1 levels in miR-9-5p-overexpressing cells (Fig. 2g). Overall, these results indicate that miR-9-5p overexpression leads to increased migration and invasiveness, EMT induction and increased activation of Akt/β-catenin signalling.

### Overexpression of miR-9-5p promotes tumour formation in vivo

**Fig. 3:**
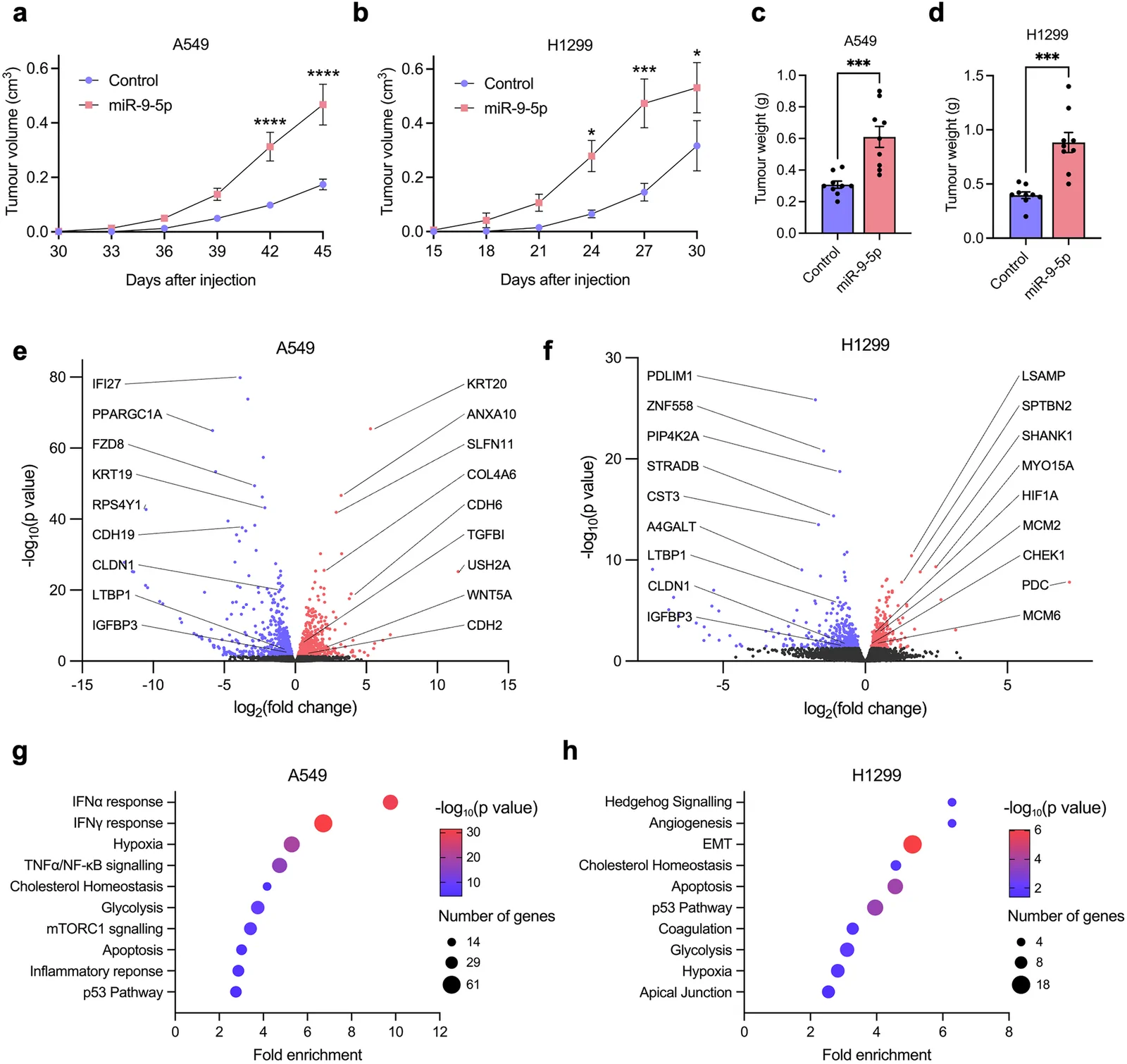
Upregulation of miR-9-5p is oncogenic in vivo. Tumour growth over time with (**a**) A549 or (**b**) H1299 cells, *n* = 9 for each condition. Results are presented as mean ± SEM *p < 0.05, ***p < 0.001, ****p < 0.0001) (two-way ANOVA with Bonferroni’s multiple comparison test), *n* = 9 for each condition. Tumour weight of tumour xenografts of (**c**) A549 and (**d**) H1299 cells. Results are presented as mean ± SEM (two-tailed unpaired Student’s t test, ***p < 0.001, *n* = 9 for each condition. Volcano plot of differentially expressed genes in (**e**) A549 and (**f**) H1299 miR-9-5p tumours, determined by DESeq2 analysis. Significant hits (*n* = 4 biological samples for each condition, *p*-adjusted < 0.05) are depicted as red for upregulated and blue for downregulated genes. Pathway enrichment analysis of downregulated genes in (**g**) A549 and (**h**) H1299 miR-9-5p tumours using MSigDB Hallmark gene sets. FDR-adjusted *p*-values are shown.

To investigate the role of miR-9-5p in lung tumourigenesis in vivo, we generated tumour xenograft models using A549 and H1299 cells overexpressing miR-9-5p (Supplementary Fig. 2b, c) in immunocompromised NOD Scid Gamma (NSG) mice. Cells were injected bilaterally in the flanks and allowed to grow for 4-6 weeks. The growth of the miR-9-5p overexpressing tumours was significantly faster than the controls (Fig. 3a, b), which is also reflected in the increased tumour weight at the study endpoint (Fig. 3c, d) (Supplementary Fig. 3a). Persistence of miR-9-5p overexpression in the tumour xenografts was also confirmed by RT-qPCR analysis of the endpoint tumours (Supplementary Fig. 3b, c).

Next, in order to identify miR-9-5p-dependent changes in gene expression in the tumour samples, mRNA sequencing was performed (Tables S2, S3), highlighting several differentially expressed genes (DEGs) (Fig. 3e, f) (Supplementary Fig. 4a-d). Pathway enrichment analysis of significantly downregulated genes using the MSigDB Hallmark database identified hypoxia, glycolysis, cholesterol homeostasis, p53 signalling and apoptosis in both cell lines. In addition, IFN-α/-γ and TNFα/NF-κB-mediated inflammatory response pathways were enriched in A549 cells, while in H1299 cells, altered pathways included EMT and Hedgehog signalling, an important cell-to-cell communication pathway [43] (Fig. 3g, h). Importantly, IFN-α/-γ and NF-κB signalling are linked to EMT [44, 45]. These results demonstrate that miR-9-5p overexpression accelerates lung tumour growth in vivo and drives gene expression changes affecting several key oncogenic pathways.

**Fig. 4:**
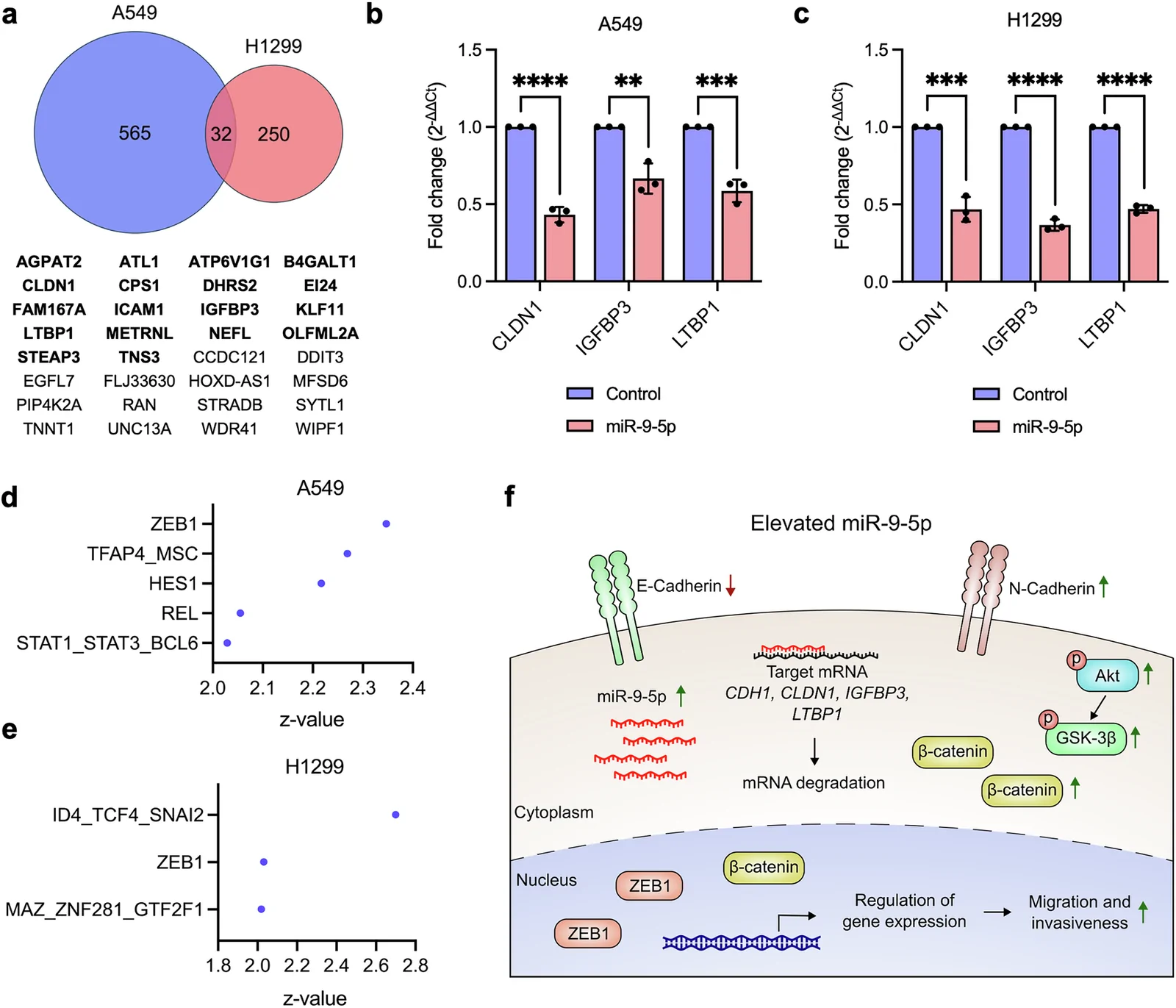
Upregulation of miR-9-5p results in widespread transcriptomics changes in lung cancer cells. **a** Venn diagram depicting the common downregulated genes in A549 and H1299 tumours. Out of 32 common genes, 18 genes are miR-9-5p predicted targets, highlighted in bold. **b**, **c** RT-qPCR analysis of *CLDN1*, *LTBP1* and *IGFBP3* expression in A549 and H1299 miR-9-5p samples. *GAPDH* was used as an internal control. Graphs show mean ± SEM, **p < 0.01, ***p < 0.001, ****p < 0.0001 (two-tailed unpaired Student’s t test), *n* = 3. **d**, **e** Significantly enriched EMT related TFs identified by transcription factor motif enrichment analysis of differentially expressed genes with ISMARA in (**d**) A549 and (**e**) H1299 miR-9-5p tumours. **f** Schematic representation of the mechanism through which miR-9-5p promotes oncogenesis in lung cancer cells.

Analysis of downregulated genes in each cell line showed that miR-9-5p overexpression resulted in significant gene downregulation in both xenograft models, with only a small subset of targets shared between A549 and H1299. Among the 32 common downregulated genes of both cell lines, 18 genes were predicted miR-9-5p targets according to TarBase v.9.0 (Fig. 4a), including the ΕΜT-implicated genes *CLDN1*, *LTBP1* and *IGFBP3*, which were further validated by RT-qPCR (Fig. 4b, c).

Transcription factor motif enrichment analysis on the differentially expressed genes was carried out by Integrated Motif Activity Response Analysis (ISMARA) (Supplementary Fig. 4e, f) [46], showing enrichment in EMT-related TFs, such as *ZEB1, TFAP4, HES1, REL* and *STAT3* in A549 miR-9-5p overexpressing cells (Fig. 4d), and *ZEB1, SNAI2* and *ZNF281* in H1299 cells (Fig. 4e). Importantly these EMT TFs repress E-cadherin transcription and are linked to NF-κB signalling [44, 45], and HES1 is a known target of miR-9-5p involved in neurogenesis [47]. Notably, the miR-9-5p seed motif was highly enriched in the A549 miR-9-5p overexpressing cells, validating the regulatory effect of miR-9-5p on its target genes in this model (Supplementary Fig. 4e).

Overall, our study identifies miR-9-5p as a critical mediator of NSCLC progression, downstream of IKKα loss. Upregulation of miR-9-5p increased migration, invasion and EMT, stabilised β-catenin and activated canonical Wnt signalling through Akt-dependent inhibition of GSK-3β. In vivo, overexpression of miR-9-5p accelerated tumour growth and caused extensive transcriptomic reprogramming. Collectively, these findings establish miR-9-5p as an oncogenic driver in NSCLC and highlight it as a potential therapeutic target to limit tumour progression (Fig. 4f).

## Discussion

IKKα has several NF-κΒ-dependent and independent roles, acting as a tumour suppressor in lung cancer [4, 8, 18–20]. However, the precise role of IKKα in lung cancer is still underexplored and further research is required to elucidate the molecular mechanisms of the tumour suppressive action of IKKα. In this study, we identified several differentially expressed miRNAs following IKKα silencing. Furthermore, most IKKα^KD^ hits did not correlate with IKKβ^KD^ hits, suggesting IKK complex-independent regulation (Fig. 1b). Pathway enrichment analysis showed the altered miRNA-targeted genes implicated in the cell cycle, p53 signalling and cellular senescence signalling, which are all highly dysregulated in lung cancer [48] (Supplementary Fig. 1c). The most highly upregulated miRNA in IKKα^KD^ cells was the oncogenic, NF-κB-regulated miRNA, miR-9-5p [5, 21]. Expression of miR-9-5p is induced by pro-inflammatory stimulation in human monocytes [24, 49]. Furthermore, miR-9-5p targets *NFKB1*, which encodes the p105/p50 precursor subunit and renders lung cancer cells sensitive to ionising radiation [49], suggesting that miR-9-5p is part of a negative feedback loop mechanism regulating the inflammatory responses [5, 24].

We next assessed whether IKKα regulates miR-9-5p expression through canonical or non-canonical NF-κB signalling. Constitutive activation of IKKβ increased miR-9-5p levels in A549 cells, while inflammatory stimulation with TNFα or treatment with the IKK inhibitor sulfasalazine did not alter miR-9-5p levels (Fig. 1d–f). These findings suggest that acute activation or inhibition of the canonical pathway do not affect miR-9-5p expression; however, sustained activation through IKKβ can promote miR-9-5p expression. This observation is consistent with cancer-associated NF-κB signalling, where a sustained activation, rather than transient pathway activation, drives a pro-tumourigenic transcriptional program [50].

As IKKα is a key regulator of the non-canonical NF-κB pathway, we assessed miR-9-5p expression following p52 depletion. Knockdown of p52 reduced miR-9-5p levels, suggesting that p52 positively regulates miR-9-5p expression (Fig. 1g, h). This contrasts with the increase in miR-9-5p observed upon IKKα depletion, indicating that IKKα is unlikely to regulate miR-9-5p through p52 and may act through a distinct mechanism. To further explore the mechanism of IKKα-mediated regulation, we re-expressed a kinase-dead IKKα mutant (K44M) in IKKα knockout cells. This resulted in partial rescue of miR-9-5p levels, suggesting that the kinase activity may contribute to the regulation of miR-9-5p (Fig. 1i, j). However, the incomplete rescue indicates that additional mechanisms may be involved. Consistent with this, IKKα has previously been reported to mediate transcriptional regulation of target genes through kinase-independent roles [51]. Collectively, these findings indicate that IKKα negatively regulates miR-9-5p in lung cancer cells, while other NF-κB pathway components, including IKKβ and p52, promote its expression in this context.

In lung cancer, miR-9-5p is often overexpressed and associates with increased metastasis [31, 52], however the complete mechanism is yet to be elucidated, therefore, we decided to further study the effects of miR-9-5p in NSCLC development. We found that LUAD displayed the highest upregulation of miR-9-5p in TCGA cancer samples compared to normal tissue among the different forms of cancer (Fig. 1k). Pathway enrichment analysis of the top experimentally supported target genes of miR-9-5p pointed to terms such as adherens junction and focal adhesion pathways (Fig. 1l), and experimentally, miR-9-5p upregulation significantly increased cell migration and invasion, linking miR-9-5p to an enhanced EMT phenotype in NSCLC cells (Fig. 3b, e). These results agree with previously published studies, where miR-9-5p promotes cell invasion and EMT in diverse cancer types [21, 53–57].

In many cancers, including lung cancer, loss of E-cadherin is a critical marker of EMT [58]. E-cadherin is a miR-9-5p target in breast cancer [53], colon cancer [21, 54], cervical cancer [59] and osteosarcoma [56], Here, we show that E-cadherin protein levels decrease in A549 miR-9-5p overexpressing cells (Fig. 2b, e). While E-cadherin levels decreased, N-cadherin and vimentin protein levels were elevated (Fig. 2f). The switch from E-cadherin to N-cadherin expression in epithelial cells is highly associated with EMT and with an invasive phenotype [45, 60]. Furthermore, vimentin upregulation promotes cell migration by supporting cytoskeletal reorganisation [61] and is associated with poor prognosis and metastasis in several cancers, including lung cancer [62]. In addition, miR-9-5p overexpressing cells showed higher levels of active Akt1 and β-catenin, while GSK-3β, a negative regulator of β-catenin [63], was phosphorylated and inactivated, indicating altogether upregulation of Akt1/GSK-3β/β-catenin pathway (Fig. 2g). Wnt signalling regulates cell fate determination and organogenesis during embryonic development, and is often activated in cancer, where it promotes proliferation and migration. β-catenin is the main downstream effector of the pathway, and when activated, it translocates to the nucleus where it induces expression of EMT-related genes [64]. Therefore, these results suggest that upregulation of miR-9-5p promotes enhanced EMT characteristics possibly through the Wnt pathway.

Our in vivo models showed accelerated tumour growth with miR-9-5p overexpression in both cell types, underscoring its potential as a key promoter of NSCLC malignancy. Other studies have demonstrated the tumour-promoting role of miR-9-5p in vivo in different cancer types, such as mouse glioma [65], Hodgkin’s lymphoma [66], and breast cancer [53]. In breast cancer, miR-9-5p promotes growth, angiogenesis and metastasis by targeting *CDH1*. E-cadherin downregulation by miR-9-5p also activates β-catenin signalling, upregulating VEGF-A expression and increasing angiogenesis, while overexpression of miR-9-5p in non-metastatic breast tumour cells enables them to form lung micrometastases in mice [53]. This current study provides in vivo evidence that elevated miR-9-5p expression both enhances tumour growth and orchestrates extensive transcriptomic changes consistent with a more aggressive phenotype. Through RNA sequencing of the resulting tumour xenografts, we showed that the upregulation miR-9-5p led to the dysregulation of genes involved in EMT and NF-κB TF signalling [3, 5, 44, 45, 67]. The limited overlap of downregulated genes between the two cell lines likely reflects their distinct genetic and molecular backgrounds, which influence baseline gene expression, pathway activation, and miRNA target availability.

Mechanistically, our study supports a model where miR-9-5p upregulation contributes to EMT and tumour progression partially by stabilising β-catenin through targeting *CDH1*, and by Akt-mediated inhibition of GSK-3β, inducing canonical Wnt signalling. This is further supported by the TF motif enrichment analysis of the DEGs, which showed activation of the TFs *ZEB1, SNAI2*, *REL*, *STAT3*, and *TFAP4*, which are well-established regulators of EMT and tumour plasticity [44, 68]. Importantly, ZEB1 and Slug directly inhibit *CDH1* transcription. These crucial EMT regulators are direct or indirect *CDH1* transcriptional repressors and downstream NF-κB targets and induce EMT, migration and invasion [69, 70].

Furthermore, the miR-9-5p seed motif itself was highly enriched within the transcriptomic landscape of A549 xenografts, suggesting a direct regulatory role of this miRNA on a substantial fraction of the dysregulated genes. Importantly, several of these genes, including *CLDN1*, *LTBP1*, and *IGFBP3*, have established roles in maintaining epithelial integrity and suppressing metastasis, suggesting their downregulation may be critical for the invasive phenotype conferred by miR-9-5p. Claudin-1 (*CLDN1*) is a tight junction protein that suppresses metastasis in LUAD, while low levels of claudin-1 are associated with poorer prognosis [71, 72]. Latent TGF-β-binding protein 1 (LTBP1) is a large extracellular matrix protein and regulator of TGF-β bioavailability [73]. In esophageal squamous cell carcinoma, LTBP1 promotes EMT via TGF-β signalling and high levels correlate with lymph node metastasis [74], however, studies in other cancer types show conflicting roles [75]. Insulin‑like growth factor binding protein 3 (IGFBP3) acts as the main carrier of insulin-like growth factor (IGF) in blood circulation. In lung cancer, IGFBP3 inhibits MMP-1 release, reducing invasiveness, while increased plasma IGFBP3 correlates with favourable diagnosis [76]. Therefore, the pro-invasive phenotype of miR-9-5p overexpressing cells could be, in part, due to the regulation of these target genes, as well as the induction of EMT transcription factors.

## Conclusion

This study aimed to explore the miRNAs regulated by IKKα to better understand its downstream signalling and tumour suppressor activity in NSCLC. Our results demonstrate that IKKα negatively regulates the expression of miR-9-5p, a tumour promoting miRNA in lung cancer. We showed that upregulation of miR-9-5p promotes EMT by downregulating E-cadherin and by activating the Akt1/GSK-3β/β-catenin pathway. Phenotypically, overexpression of miR-9-5p increased cell migration and invasion in vitro and tumour growth in vivo. Transcriptomics analysis of the tumour xenografts revealed widespread changes in gene expression, favouring tumour growth and EMT. Overall, these data identify a novel IKKα-regulated miRNA and demonstrate how miR-9-5p contributes to EMT and transcriptomic changes in lung cancer.

## Methods

### Cell culture

A549, NCI-H1299, Phoenix and HEK293T cells were obtained from American Type Culture Collection (ATCC). Cells were recently authenticated and regularly tested for contamination. A549 and H1299 cells were cultured in low glucose DMEM (Sigma #D6046-500ML) and Phoenix and HEK293T cells were cultured in high glucose DMEM (Sigma #D6429-500), all supplemented with 10% Fetal Bovine Serum (FBS) (Biowest #S181B-500), 1% Penicillin-Streptomycin (Biowest #L0022-100) and 2 mM L-Glutamine (Biowest #X0550-100). Cells were maintained in a humidified incubator, with 5% CO_2_ at 37 °C.

### Establishment of stable cell lines

To establish IKKα^KD^, IKKβ^KD^ and p52^KD^ cell lines, the pSUPER.retro vector was used containing shIKKα, shIKKβ or shp52, and non-targeting shRNA-containing pSUPER.retro plasmid was used to generate control cells [77–80]. To establish the A549 IKKβ^CΑ^ (constitutively active) cell line, pCLXSN-IKKβ^CA^ carrying a constitutively active T-loop mutant form of human IKKβ was used [81]. pCLXSN-ires-GFP was used as a control. Cells were selected with 1 μg/ml puromycin for 1 week.

To generate miR-9-5p overexpressing cell lines, the lentiviral vectors Twl-PGKgfp-H1 and Twl-PGKgfp-H1-miR-9-5p were used [39]. To generate the A549 IKKα^KO^ cell line, the lenti-vector pLentiCRISPR v2 containing IKKα sgRNA was used, and the empty vector was used as a control. The vectors were kindly gifted by Dr Ana O’Loghlen [82]. Following lentiviral transduction, the cells were selected with puromycin and single clone colonies were generated. For the establishment of kinase-dead IKKα cell lines (IKKα^K44M^), the retro-vector pBIN-IKKαK44M-HA, expressing a mouse IKKα with a lysine 44 to methionine mutation, was used in A549 IKKα^KO^ cells and neomycin was used for selection [83]. Retroviral particles were produced by Phoenix cells as previously described [8, 79]. Lentiviral particles were produced by HEK293T cells.

### Cell treatments

For canonical NF-κB pathway activation, cells were treated with 10 ng/ml TNF-α (MedChemExpress #HY-P7058) for 2 h for western blot analysis and 24 h for RT-qPCR. For the inhibition of the canonical NF-κB pathway, cells were treated with 2 μM sulfasalazine (MedChemExpress #HY-14655) for 2 h for western blot analysis and 24 h for RT-qPCR.

### Nanostring microRNA analysis

NanoString nCounter technology was used to examine miRNA expression [26]. 100 ng of total RNA for each sample was used, and RNA quality was assessed using a Bioanalyser. Full details are included in the supplementary information.

### Real time quantitative polymerase chain reaction (RT-qPCR)

Cells were lysed using the QIAzol Lysis Reagent (Qiagen #1023537) following the manufacturer’s instructions. Total RNA was extracted using the miRNeasy Mini Kit (Qiagen #217004). For cDNA synthesis, reverse transcription reaction was carried out using the miRCURY LNA RT Kit (Qiagen #339340) according to manufacturer’s instructions. RT-qPCR reactions were performed using the miRCURY LNA SYBR Green PCR Kit (Qiagen #339346). Fold change was calculated using the 2^-ΔΔCt^ method.

### Cell lysis and western blotting

Cell lysates were prepared using RIPA buffer for 60 min at 4 °C. Cell lysates were then centrifuged for 15 min at 13,000 × g at 4 °C and the pellets were discarded. 40 μg of protein samples were analysed by SDS-PAGE followed by immunoblotting. Immunoblots were developed with an ECL detection kit (BioRad #170–5061) using the Imager Molecular Imager® Chemi Doc™ XRS (BioRad).

### Wound healing assay

2 × 10^5^ cells were seeded per well of a 24-well plate. After 24 h, a scratch was created using a sterile pipette tip. The cells were then washed in PBS and allowed to grow in serum-free media for 24 h. Images were taken at the beginning and end of the growth period and ImageJ was used to analyse wound closure.

### Matrigel invasion assay

Matrigel (Corning #356230; 80 μl, 1:5 in serum-free media) was added to transwell inserts (24 well, 8 μm pore size, Sarstedt #83.3932.800) and allowed to solidify at 37 °C for 2 h. 4 × 10^4^ cells were seeded in 300 μl serum-free media into the inserts, with 1 ml of media containing 20% FBS in the wells below. After 48 h, the cells were washed with PBS, fixed in 4% PFA for 15 min, then stained with 1% crystal violet in 20% ethanol for 20 min. Inserts were washed thoroughly and 10 representative images were captured per insert using an EVOS microscope. Infiltrating cells were quantified with ImageJ, based on mean intensity.

### Xenograft mouse models

4 × 10^6^ cells in 200 μl of PBS were subcutaneously inoculated into male immunocompromised NSG (NOD-SCID-IL2Rgamma) mice aged 5 weeks. Each animal was inoculated bilaterally, with modified cells (left side for control Twl-PGKgfp-H1 A549 and H1299 and right side for TwI-miR-9-5p A549 and H1299 cells, *n* = 9 per cell line), therefore, randomisation to experimental groups was not applicable. Mice were sacrificed after 4 and 6 weeks for H1299 and A549, respectively after cell inoculation. The isolated tumours were weighed and immediately snap-frozen in liquid nitrogen, then stored at −80 °C for RNA isolation.

### RNA sequencing

For total RNA isolation from tumours grown from control and miR-9-5p A549 and H1299 cells, mortar and pestle disruption with liquid nitrogen was used to grind tissue into fine powder. For each sample, 50 mg of tissue was lysed with 700 μl QIAzol lysis reagent. Total RNA was extracted as previously described (*n* = 4 for each condition). RNA-Seq analysis was performed at the Greek Genome Centre in BRFAA. Differential gene expression analysis was performed using DESeq2 [84–87], followed by pathway enrichment analysis using ShinyGO [88]. Full details are provided in the supplementary information.

### Statistical analysis and experimental design

GraphPad Prism 10 software was used for statistical data analysis. A two-tailed Student’s t-test or analysis of variance (ANOVA) was performed as appropriate, and *p values *<0.05; **p < 0.01; ***p < 0.001; ****p < 0.0001 were considered significant. Data are presented as mean ± the standard error of the mean (SEM). Individual data points are shown where possible. Data distribution was assumed to be normal and variance was similar between tested groups. Sample sizes were selected based on previous similar studies. Investigators were not blinded while conducting animal experiments, and no animals were excluded from analysis. Statistical tests and *p*-values are included in the figure legends.

## Supplementary information

Supplementary Figures

Supplementary Information

Supplementary Table 1

Supplementary Table 2

Supplementary Table 3

Original uncropped western blots

## Data Availability

All raw and processed RNA sequencing data generated in this study are available in GEO under accession GSE327377. All remaining relevant data are contained within the main article and its Supplementary Files.

## References

[1] Bray F, Laversanne M, Sung H, Ferlay J, Siegel RL, Soerjomataram I, et al. Global cancer statistics 2022: GLOBOCAN estimates of incidence and mortality worldwide for 36 cancers in 185 countries. CA Cancer J Clin. 2024;74:229–63. 38572751 10.3322/caac.21834

[2] Perkins ND. Integrating cell-signalling pathways with NF-kappaB and IKK function. Nat Rev Mol Cell Biol. 2007;8:49–62. 17183360 10.1038/nrm2083

[3] Perkins ND. The diverse and complex roles of NF-kappaB subunits in cancer. Nat Rev Cancer. 2012;12:121–32. 22257950 10.1038/nrc3204

[4] Li X, Hu Y Attribution of NF-kappaB activity to CHUK/IKKalpha-involved carcinogenesis. Cancers. 2021;13. 10.3390/cancers13061411 PMC8003426 33808757

[5] Markopoulos GS, Roupakia E, Tokamani M, Alabasi G, Sandaltzopoulos R, Marcu KB, et al. Roles of NF-kappaB signaling in the regulation of miRNAs impacting on inflammation in cancer. Biomedicines. 2018;6. 10.3390/biomedicines6020040 PMC6027290 29601548

[6] Takahashi H, Ogata H, Nishigaki R, Broide DH, Karin M. Tobacco smoke promotes lung tumorigenesis by triggering IKKbeta- and JNK1-dependent inflammation. Cancer Cell. 2010;17:89–97. 20129250 10.1016/j.ccr.2009.12.008 PMC2818776

[7] Basseres DS, Ebbs A, Cogswell PC, Baldwin AS. IKK is a therapeutic target in KRAS-Induced lung cancer with disrupted p53 activity. Genes Cancer. 2014;5:41–55. 24955217 10.18632/genesandcancer.5 PMC4063255

[8] Chavdoula E, Habiel DM, Roupakia E, Markopoulos GS, Vasilaki E, Kokkalis A, et al. CHUK/IKK-alpha loss in lung epithelial cells enhances NSCLC growth associated with HIF up-regulation. Life Sci Alliance. 2019;2. 10.26508/lsa.201900460 PMC6892436 31792060

[9] Stathopoulos GT, Sherrill TP, Cheng DS, Scoggins RM, Han W, Polosukhin VV, et al. Epithelial NF-kappaB activation promotes urethane-induced lung carcinogenesis. Proc Natl Acad Sci USA. 2007;104:18514–9. 18000061 10.1073/pnas.0705316104 PMC2141808

[10] Meylan E, Dooley AL, Feldser DM, Shen L, Turk E, Ouyang C, et al. Requirement for NF-kappaB signalling in a mouse model of lung adenocarcinoma. Nature. 2009;462:104–7. 19847165 10.1038/nature08462 PMC2780341

[11] Roupakia E, Chavdoula E, Karpathiou G, Vatsellas G, Chatzopoulos D, Mela A, et al. Canonical NF-kappaB promotes lung epithelial cell tumour growth by downregulating the metastasis suppressor CD82 and enhancing epithelial-to-mesenchymal cell transition. Cancers. 2021;13. 10.3390/cancers13174302 PMC8428346 34503110

[12] Basseres DS, Ebbs A, Levantini E, Baldwin AS. Requirement of the NF-kappaB subunit p65/RelA for K-Ras-induced lung tumorigenesis. Cancer Res. 2010;70:3537–46. 20406971 10.1158/0008-5472.CAN-09-4290 PMC2862109

[13] Fernandez-Majada V, Aguilera C, Villanueva A, Vilardell F, Robert-Moreno A, Aytes A, et al. Nuclear IKK activity leads to dysregulated notch-dependent gene expression in colorectal cancer. Proc Natl Acad Sci USA. 2007;104:276–81. 17190815 10.1073/pnas.0606476104 PMC1765449

[14] Anest V, Cogswell PC, Baldwin AS Jr. IkappaB kinase alpha and p65/RelA contribute to optimal epidermal growth factor-induced c-fos gene expression independent of IkappaBalpha degradation. J Biol Chem. 2004;279:31183–9. 15155743 10.1074/jbc.M404380200

[15] Dong W, Li Y, Gao M, Hu M, Li X, Mai S, et al. IKKalpha contributes to UVB-induced VEGF expression by regulating AP-1 transactivation. Nucleic Acids Res. 2012;40:2940–55. 22169952 10.1093/nar/gkr1216 PMC3326327

[16] Antonia RJ, Hagan RS, Baldwin AS. Expanding the view of IKK: new substrates and new biology. Trends Cell Biol. 2021;31:166–78. 33422358 10.1016/j.tcb.2020.12.003

[17] Yamamoto Y, Verma UN, Prajapati S, Kwak YT, Gaynor RB. Histone H3 phosphorylation by IKK-alpha is critical for cytokine-induced gene expression. Nature. 2003;423:655–9. 12789342 10.1038/nature01576

[18] Song NY, Zhu F, Wang Z, Willette-Brown J, Xi S, Sun Z, et al. IKKalpha inactivation promotes Kras-initiated lung adenocarcinoma development through disrupting major redox regulatory pathways. Proc Natl Acad Sci USA. 2018;115:E812–E21. 29311298 10.1073/pnas.1717520115 PMC5789942

[19] Song NY, Li X, Ma B, Willette-Brown J, Zhu F, Jiang C, et al. IKKalpha-deficient lung adenocarcinomas generate an immunosuppressive microenvironment by overproducing Treg-inducing cytokines. Proc Natl Acad Sci USA. 2022;119. 10.1073/pnas.2120956119 PMC8833198 35121655

[20] Xiao Z, Jiang Q, Willette-Brown J, Xi S, Zhu F, Burkett S, et al. The pivotal role of IKKalpha in the development of spontaneous lung squamous cell carcinomas. Cancer Cell. 2013;23:527–40. 23597566 10.1016/j.ccr.2013.03.009 PMC3649010

[21] Markopoulos GS, Roupakia E, Tokamani M, Chavdoula E, Hatziapostolou M, Polytarchou C, et al. A step-by-step microRNA guide to cancer development and metastasis. Cell Oncol. 2017;40:303–39. 10.1007/s13402-017-0341-9 PMC13001585 28748501

[22] Hobert O. Gene regulation by transcription factors and microRNAs. Science. 2008;319:1785–6. 18369135 10.1126/science.1151651

[23] Ma X, Becker Buscaglia LE, Barker JR, Li Y. MicroRNAs in NF-kappaB signaling. J Mol Cell Biol. 2011;3:159–66. 21502305 10.1093/jmcb/mjr007 PMC3104013

[24] Boldin MP, Baltimore D. MicroRNAs, new effectors and regulators of NF-kappaB. Immunol Rev. 2012;246:205–20. 22435557 10.1111/j.1600-065X.2011.01089.x

[25] Li T, Morgan MJ, Choksi S, Zhang Y, Kim YS, Liu ZG. MicroRNAs modulate the noncanonical transcription factor NF-kappaB pathway by regulating expression of the kinase IKKalpha during macrophage differentiation. Nat Immunol. 2010;11:799–805. 20711193 10.1038/ni.1918 PMC2926307

[26] Geiss GK, Bumgarner RE, Birditt B, Dahl T, Dowidar N, Dunaway DL, et al. Direct multiplexed measurement of gene expression with color-coded probe pairs. Nat Biotechnol. 2008;26:317–25. 18278033 10.1038/nbt1385

[27] Markopoulos GS, Roupakia E, Tokamani M, Vartholomatos G, Tzavaras T, Hatziapostolou M, et al. Senescence-associated microRNAs target cell cycle regulatory genes in normal human lung fibroblasts. Exp Gerontol. 2017;96:110–22. 28658612 10.1016/j.exger.2017.06.017

[28] Foutadakis S, Soureas K, Roupakia E, Besta S, Avgeris M, Kolettas E. Identification of Oncogene-Induced Senescence-Associated MicroRNAs. Methods Mol Biol. 2025;2906:189–213. 40082357 10.1007/978-1-0716-4426-3_11

[29] Chang L, Xia J. MicroRNA Regulatory Network Analysis Using miRNet 2.0. Methods Mol Biol. 2023;2594:185–204. 36264497 10.1007/978-1-0716-2815-7_14

[30] Skoufos G, Kakoulidis P, Tastsoglou S, Zacharopoulou E, Kotsira V, Miliotis M, et al. TarBase-v9.0 extends experimentally supported miRNA-gene interactions to cell-types and virally encoded miRNAs. Nucleic Acids Res. 2024;52:D304–D10. 37986224 10.1093/nar/gkad1071 PMC10767993

[31] Xu T, Liu X, Han L, Shen H, Liu L, Shu Y. Up-regulation of miR-9 expression as a poor prognostic biomarker in patients with non-small cell lung cancer. Clin Transl Oncol. 2014;16:469–75. 24019037 10.1007/s12094-013-1106-1

[32] Li G, Wu F, Yang H, Deng X, Yuan Y. MiR-9-5p promotes cell growth and metastasis in non-small cell lung cancer through the repression of TGFBR2. Biomed Pharmacother. 2017;96:1170–8. 29239816 10.1016/j.biopha.2017.11.105

[33] Han L, Wang W, Ding W, Zhang L. MiR-9 is involved in TGF-beta1-induced lung cancer cell invasion and adhesion by targeting SOX7. J Cell Mol Med. 2017;21:2000–8. 28266181 10.1111/jcmm.13120 PMC5571535

[34] Zhu K, Lin J, Chen S, Xu Q miR-9-5p promotes lung adenocarcinoma cell proliferation, migration and invasion by targeting ID4. Technol Cancer Res Treat. 2021;20: 15330338211048592. 10.1177/15330338211048592 PMC8564129 34723712

[35] Wahl C, Liptay S, Adler G, Schmid RM. Sulfasalazine: a potent and specific inhibitor of nuclear factor kappa B. J Clin Invest. 1998;101:1163–74. 9486988 10.1172/JCI992 PMC508669

[36] Weber CK, Liptay S, Wirth T, Adler G, Schmid RM. Suppression of NF-kappaB activity by sulfasalazine is mediated by direct inhibition of IkappaB kinases alpha and beta. Gastroenterology. 2000;119:1209–18. 11054378 10.1053/gast.2000.19458

[37] Wong NW, Chen Y, Chen S, Wang X. OncomiR: an online resource for exploring pan-cancer microRNA dysregulation. Bioinformatics. 2018;34:713–5. 29028907 10.1093/bioinformatics/btx627 PMC5860608

[38] Tastsoglou S, Skoufos G, Miliotis M, Karagkouni D, Koutsoukos I, Karavangeli A, et al. DIANA-miRPath v4.0: expanding target-based miRNA functional analysis in cell-type and tissue contexts. Nucleic Acids Res. 2023;51:W154–W9. 37260078 10.1093/nar/gkad431 PMC10320185

[39] Mingardi J, La Via L, Tornese P, Carini G, Trontti K, Seguini M, et al. miR-9-5p is involved in the rescue of stress-dependent dendritic shortening of hippocampal pyramidal neurons induced by acute antidepressant treatment with ketamine. Neurobiol Stress. 2021;15:100381. 34458512 10.1016/j.ynstr.2021.100381 PMC8379501

[40] Ozawa M, Baribault H, Kemler R. The cytoplasmic domain of the cell adhesion molecule uvomorulin associates with three independent proteins structurally related in different species. EMBO J. 1989;8:1711–7. 2788574 10.1002/j.1460-2075.1989.tb03563.x PMC401013

[41] Ozawa M, Kemler R. Altered cell adhesion activity by pervanadate due to the dissociation of alpha-catenin from the E-cadherin.catenin complex. J Biol Chem. 1998;273:6166–70. 9497337 10.1074/jbc.273.11.6166

[42] Nagafuchi A. Molecular architecture of adherens junctions. Curr Opin Cell Biol. 2001;13:600–3. 11544029 10.1016/s0955-0674(00)00257-x

[43] Cong G, Zhu X, Chen XR, Chen H, Chong W. Mechanisms and therapeutic potential of the hedgehog signaling pathway in cancer. Cell Death Discov. 2025;11:40. 39900571 10.1038/s41420-025-02327-w PMC11791101

[44] Markopoulos GS, Roupakia E, Marcu KB, Kolettas E. Epigenetic regulation of inflammatory cytokine-induced epithelial-to-mesenchymal cell transition and cancer stem cell generation. Cells. 2019;8. 10.3390/cells8101143 PMC6829508 31557902

[45] Zhang CX, Huang RY, Sheng G, Thiery JP. Epithelial-mesenchymal transition. Cell. 2025;188:5436–86. 41043405 10.1016/j.cell.2025.08.033

[46] Balwierz PJ, Pachkov M, Arnold P, Gruber AJ, Zavolan M, van Nimwegen E. ISMARA: automated modeling of genomic signals as a democracy of regulatory motifs. Genome Res. 2014;24:869–84. 24515121 10.1101/gr.169508.113 PMC4009616

[47] Bonev B, Stanley P, Papalopulu N. MicroRNA-9 Modulates Hes1 ultradian oscillations by forming a double-negative feedback loop. Cell Rep. 2012;2:10–8. 22840391 10.1016/j.celrep.2012.05.017 PMC4103481

[48] Jha SK, De Rubis G, Devkota SR, Zhang Y, Adhikari R, Jha LA, et al. Cellular senescence in lung cancer: Molecular mechanisms and therapeutic interventions. Ageing Res Rev. 2024;97:102315. 38679394 10.1016/j.arr.2024.102315

[49] Bazzoni F, Rossato M, Fabbri M, Gaudiosi D, Mirolo M, Mori L, et al. Induction and regulatory function of miR-9 in human monocytes and neutrophils exposed to proinflammatory signals. Proc Natl Acad Sci USA. 2009;106:5282–7. 19289835 10.1073/pnas.0810909106 PMC2664036

[50] Mao H, Zhao X, Sun SC. NF-kappaB in inflammation and cancer. Cell Mol Immunol. 2025;22:811–39. 40562870 10.1038/s41423-025-01310-w PMC12310982

[51] Olivotto E, Otero M, Astolfi A, Platano D, Facchini A, Pagani S, et al. IKKalpha/CHUK regulates extracellular matrix remodeling independent of its kinase activity to facilitate articular chondrocyte differentiation. PLoS One. 2013;8:e73024. 24023802 10.1371/journal.pone.0073024 PMC3759388

[52] Volinia S, Calin GA, Liu CG, Ambs S, Cimmino A, Petrocca F, et al. A microRNA expression signature of human solid tumors defines cancer gene targets. Proc Natl Acad Sci USA. 2006;103:2257–61. 16461460 10.1073/pnas.0510565103 PMC1413718

[53] Ma L, Young J, Prabhala H, Pan E, Mestdagh P, Muth D, et al. miR-9, a MYC/MYCN-activated microRNA, regulates E-cadherin and cancer metastasis. Nat Cell Biol. 2010;12:247–56. 20173740 10.1038/ncb2024 PMC2845545

[54] Lu MH, Huang CC, Pan MR, Chen HH, Hung WC. Prospero homeobox 1 promotes epithelial-mesenchymal transition in colon cancer cells by inhibiting E-cadherin via miR-9. Clin Cancer Res. 2012;18:6416–25. 23045246 10.1158/1078-0432.CCR-12-0832

[55] Wang L, Cui M, Cheng D, Qu F, Yu J, Wei Y, et al. miR-9-5p facilitates hepatocellular carcinoma cell proliferation, migration and invasion by targeting ESR1. Mol Cell Biochem. 2021;476:575–83. 33106914 10.1007/s11010-020-03927-z

[56] Gang W, Tanjun W, Yong H, Jiajun Q, Yi Z, Hao H. Inhibition of miR-9 decreases osteosarcoma cell proliferation. Bosn J Basic Med Sci. 2020;20:218–25. 31724522 10.17305/bjbms.2019.4434 PMC7202196

[57] Drakaki A, Hatziapostolou M, Polytarchou C, Vorvis C, Poultsides GA, Souglakos J, et al. Functional microRNA high throughput screening reveals miR-9 as a central regulator of liver oncogenesis by affecting the PPARA-CDH1 pathway. BMC Cancer. 2015;15:542. 26206264 10.1186/s12885-015-1562-9 PMC4512159

[58] Sideridou M, Zakopoulou R, Evangelou K, Liontos M, Kotsinas A, Rampakakis E, et al. Cdc6 expression represses E-cadherin transcription and activates adjacent replication origins. J Cell Biol. 2011;195:1123–40. 22201124 10.1083/jcb.201108121 PMC3246883

[59] Babion I, Jaspers A, van Splunter AP, van der Hoorn IAE, Wilting SM, Steenbergen RDM. miR-9-5p exerts a dual role in cervical cancer and targets transcription factor TWIST1. Cells. 2019;9. 10.3390/cells9010065 PMC7017350 31888045

[60] Loh CY, Chai JY, Tang TF, Wong WF, Sethi G, Shanmugam MK, et al. The E-Cadherin and N-Cadherin switch in epithelial-to-mesenchymal transition: signaling, therapeutic implications, and challenges. Cells. 2019;8. 10.3390/cells8101118 PMC6830116 31547193

[61] Esue O, Carson AA, Tseng Y, Wirtz D. A direct interaction between actin and vimentin filaments mediated by the tail domain of vimentin. J Biol Chem. 2006;281:30393–9. 16901892 10.1074/jbc.M605452200

[62] Berr AL, Wiese K, Dos Santos G, Koch CM, Anekalla KR, Kidd M, et al. Vimentin is required for tumor progression and metastasis in a mouse model of non-small cell lung cancer. Oncogene. 2023;42:2074–87. 37161053 10.1038/s41388-023-02703-9 PMC10275760

[63] Liu C, Li Y, Semenov M, Han C, Baeg GH, Tan Y, et al. Control of beta-catenin phosphorylation/degradation by a dual-kinase mechanism. Cell. 2002;108:837–47. 11955436 10.1016/s0092-8674(02)00685-2

[64] Stewart DJ. Wnt signaling pathway in non-small cell lung cancer. J Natl Cancer Inst. 2014;106:djt356. 24309006 10.1093/jnci/djt356

[65] Chen X, Yang F, Zhang T, Wang W, Xi W, Li Y, et al. MiR-9 promotes tumorigenesis and angiogenesis and is activated by MYC and OCT4 in human glioma. J Exp Clin Cancer Res. 2019;38:99. 30795814 10.1186/s13046-019-1078-2 PMC6385476

[66] Leucci E, Zriwil A, Gregersen LH, Jensen KT, Obad S, Bellan C, et al. Inhibition of miR-9 de-represses HuR and DICER1 and impairs Hodgkin lymphoma tumour outgrowth in vivo. Oncogene. 2012;31:5081–9. 22310293 10.1038/onc.2012.15

[67] Hanahan D. Hallmarks of cancer: new dimensions. Cancer Discov. 2022;12:31–46. 35022204 10.1158/2159-8290.CD-21-1059

[68] Stemmler MP, Eccles RL, Brabletz S, Brabletz T. Non-redundant functions of EMT transcription factors. Nat Cell Biol. 2019;21:102–12. 30602760 10.1038/s41556-018-0196-y

[69] Wu Y, Deng J, Rychahou PG, Qiu S, Evers BM, Zhou BP. Stabilization of snail by NF-kappaB is required for inflammation-induced cell migration and invasion. Cancer Cell. 2009;15:416–28. 19411070 10.1016/j.ccr.2009.03.016 PMC2881229

[70] Storci G, Sansone P, Mari S, D’Uva G, Tavolari S, Guarnieri T, et al. TNFalpha up-regulates SLUG via the NF-kappaB/HIF1alpha axis, which imparts breast cancer cells with a stem cell-like phenotype. J Cell Physiol. 2010;225:682–91. 20509143 10.1002/jcp.22264 PMC2939957

[71] Chao YC, Pan SH, Yang SC, Yu SL, Che TF, Lin CW, et al. Claudin-1 is a metastasis suppressor and correlates with clinical outcome in lung adenocarcinoma. Am J Respir Crit Care Med. 2009;179:123–33. 18787218 10.1164/rccm.200803-456OC

[72] Paschoud S, Bongiovanni M, Pache JC, Citi S. Claudin-1 and claudin-5 expression patterns differentiate lung squamous cell carcinomas from adenocarcinomas. Mod Pathol. 2007;20:947–54. 17585317 10.1038/modpathol.3800835

[73] Robertson IB, Horiguchi M, Zilberberg L, Dabovic B, Hadjiolova K, Rifkin DB. Latent TGF-beta-binding proteins. Matrix Biol. 2015;47:44–53. 25960419 10.1016/j.matbio.2015.05.005 PMC4844006

[74] Cai R, Wang P, Zhao X, Lu X, Deng R, Wang X, et al. LTBP1 promotes esophageal squamous cell carcinoma progression through epithelial-mesenchymal transition and cancer-associated fibroblasts transformation. J Transl Med. 2020;18:139. 32216815 10.1186/s12967-020-02310-2 PMC7098101

[75] Tubita A, Menconi A, Lombardi Z, Tusa I, Esparis-Ogando A, Pandiella A, et al. Latent-Transforming Growth Factor beta-Binding Protein 1/Transforming Growth Factor beta1 Complex Drives Antitumoral Effects upon ERK5 Targeting in Melanoma. Am J Pathol. 2024;194:1581–91. 38705382 10.1016/j.ajpath.2024.03.015

[76] Kuhn H, Frille A, Petersen MA, Oberhuber-Kurth J, Hofmann L, Glaser A, et al. IGFBP3 inhibits tumor growth and invasion of lung cancer cells and is associated with improved survival in lung cancer patients. Transl Oncol. 2023;27:101566. 36257207 10.1016/j.tranon.2022.101566 PMC9583099

[77] Olivotto E, Borzi RM, Vitellozzi R, Pagani S, Facchini A, Battistelli M, et al. Differential requirements for IKKalpha and IKKbeta in the differentiation of primary human osteoarthritic chondrocytes. Arthritis Rheum. 2008;58:227–39. 18163512 10.1002/art.23211 PMC2927097

[78] Penzo M, Massa PE, Olivotto E, Bianchi F, Borzi RM, Hanidu A, et al. Sustained NF-kappaB activation produces a short-term cell proliferation block in conjunction with repressing effectors of cell cycle progression controlled by E2F or FoxM1. J Cell Physiol. 2009;218:215–27. 18803232 10.1002/jcp.21596 PMC2581928

[79] Sfikas A, Batsi C, Tselikou E, Vartholomatos G, Monokrousos N, Pappas P, et al. The canonical NF-kappaB pathway differentially protects normal and human tumor cells from ROS-induced DNA damage. Cell Signal. 2012;24:2007–23. 22750558 10.1016/j.cellsig.2012.06.010 PMC3432746

[80] Bernal-Mizrachi L, Lovly CM, Ratner L. The role of NF-kappaB-1 and NF-kappaB-2-mediated resistance to apoptosis in lymphomas. Proc Natl Acad Sci USA. 2006;103:9220–5. 16751281 10.1073/pnas.0507809103 PMC1482593

[81] Batsi C, Markopoulou S, Vartholomatos G, Georgiou I, Kanavaros P, Gorgoulis VG, et al. Chronic NF-kappaB activation delays RasV12-induced premature senescence of human fibroblasts by suppressing the DNA damage checkpoint response. Mech Ageing Dev. 2009;130:409–19. 19406145 10.1016/j.mad.2009.04.002 PMC2765928

[82] Fafian-Labora JA, O’Loghlen A. NF-kappaB/IKK activation by small extracellular vesicles within the SASP. Aging Cell. 2021;20:e13426. 34187082 10.1111/acel.13426 PMC8282244

[83] Massa PE, Li X, Hanidu A, Siamas J, Pariali M, Pareja J, et al. Gene expression profiling in conjunction with physiological rescues of IKKalpha-null cells with wild type or mutant IKKalpha reveals distinct classes of IKKalpha/NF-kappaB-dependent genes. J Biol Chem. 2005;280:14057–69. 15695520 10.1074/jbc.M414401200 PMC1226413

[84] Galaxy C. The Galaxy platform for accessible, reproducible, and collaborative data analyses: 2024 update. Nucleic Acids Res. 2024;52:W83–W94. 38769056 10.1093/nar/gkae410 PMC11223835

[85] Kim D, Paggi JM, Park C, Bennett C, Salzberg SL. Graph-based genome alignment and genotyping with HISAT2 and HISAT-genotype. Nat Biotechnol. 2019;37:907–15. 31375807 10.1038/s41587-019-0201-4 PMC7605509

[86] Anders S, Pyl PT, Huber W. HTSeq-a Python framework to work with high-throughput sequencing data. Bioinformatics. 2015;31:166–9. 25260700 10.1093/bioinformatics/btu638 PMC4287950

[87] Love MI, Huber W, Anders S. Moderated estimation of fold change and dispersion for RNA-seq data with DESeq2. Genome Biol. 2014;15:550. 25516281 10.1186/s13059-014-0550-8 PMC4302049

[88] Ge SX, Jung D, Yao R. ShinyGO: a graphical gene-set enrichment tool for animals and plants. Bioinformatics. 2020;36:2628–9. 31882993 10.1093/bioinformatics/btz931 PMC7178415

