# Supplementary material for: The IKKα-regulated microRNA miR-9-5p mediates lung cancer growth and invasiveness via CDH1/Wnt/β-catenin signalling
Source: Cell Death Discov. 2026 Jun 17;12:346. doi: 10.1038/s41420-026-03195-8 (PMC13507200; doi:10.1038/s41420-026-03195-8)

Supplementary figure 1

a

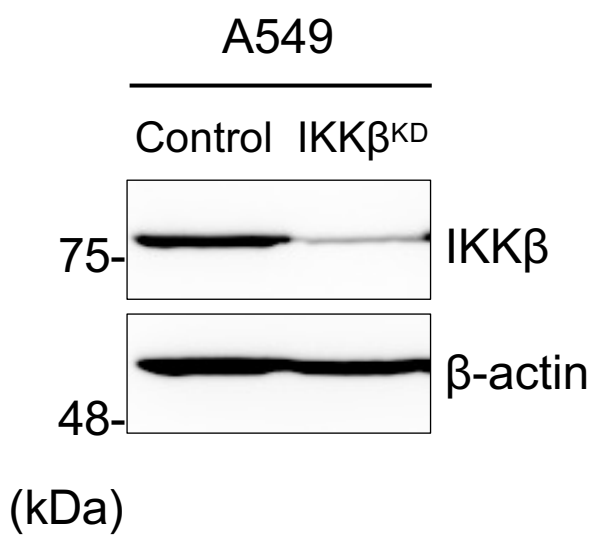

b

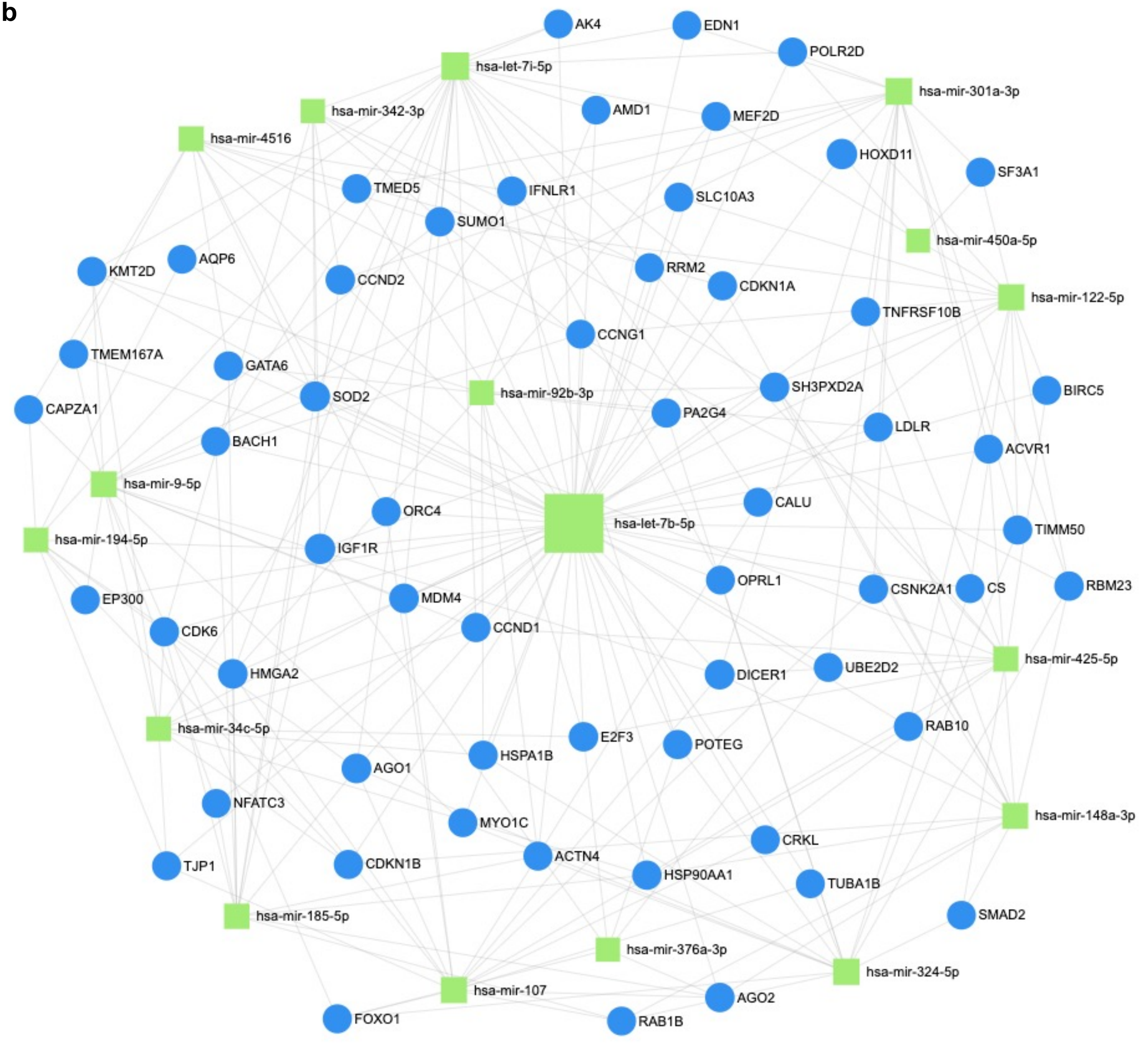

Supplementary figure 1

c

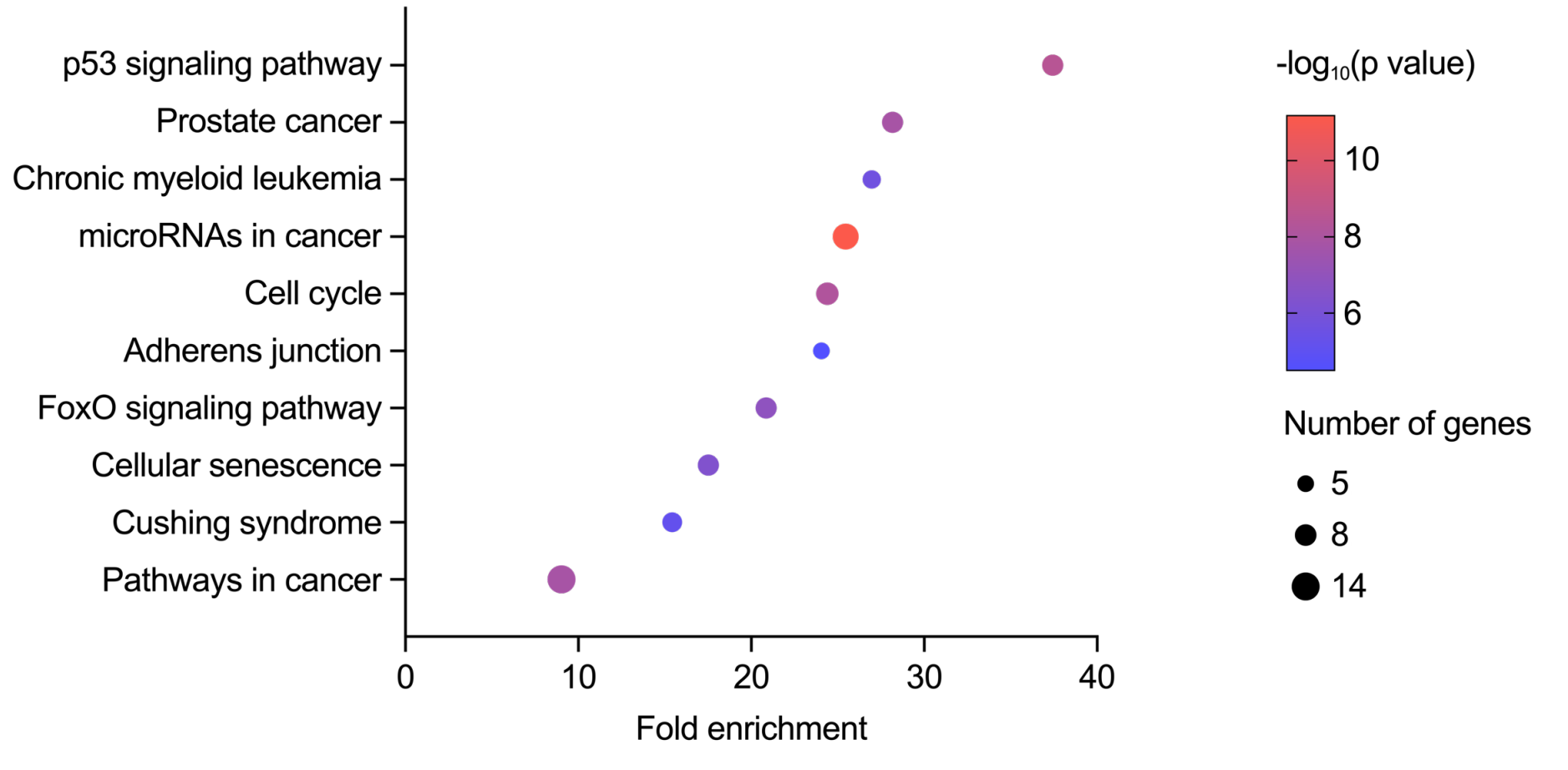

d

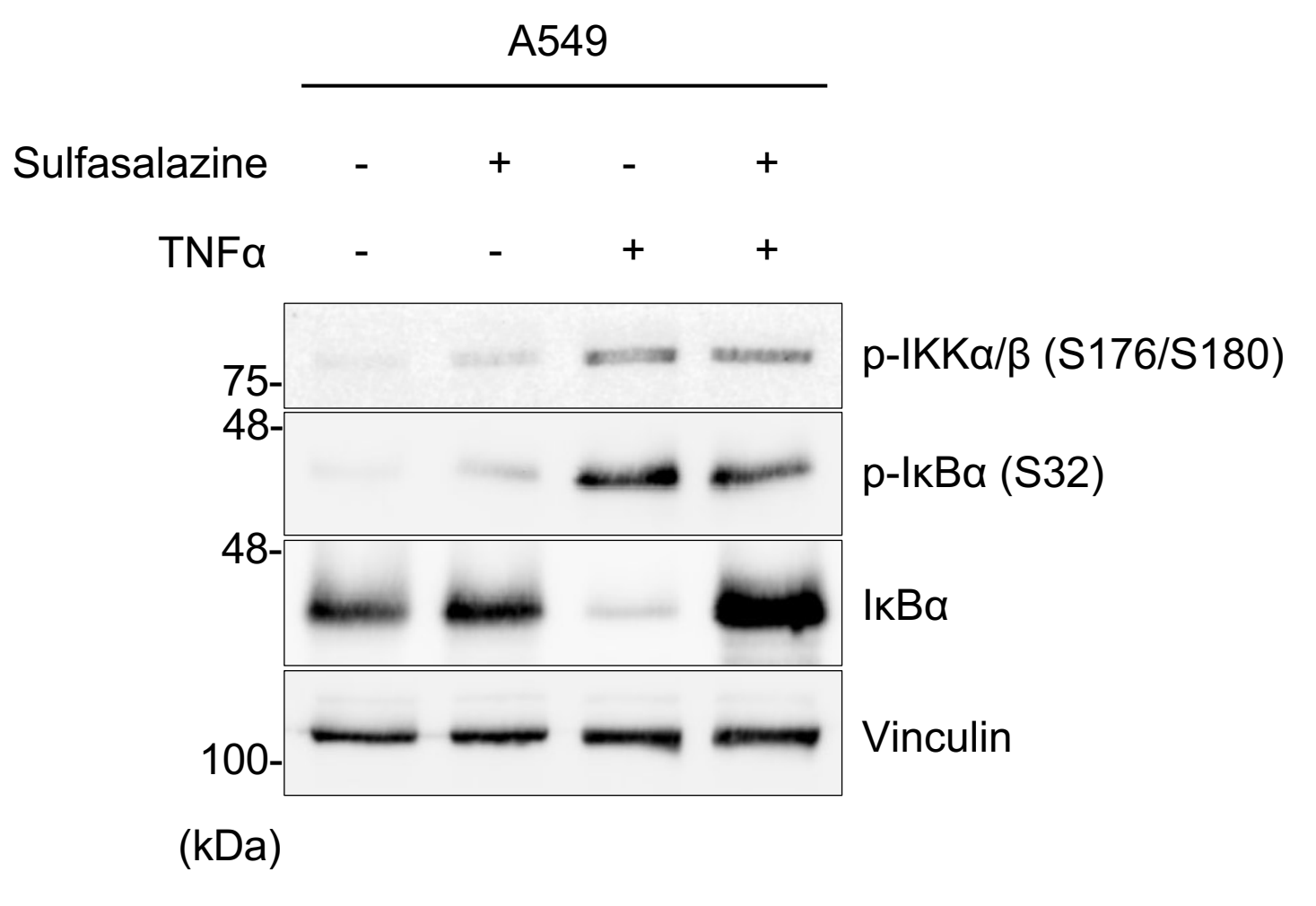

Supplementary figure 2

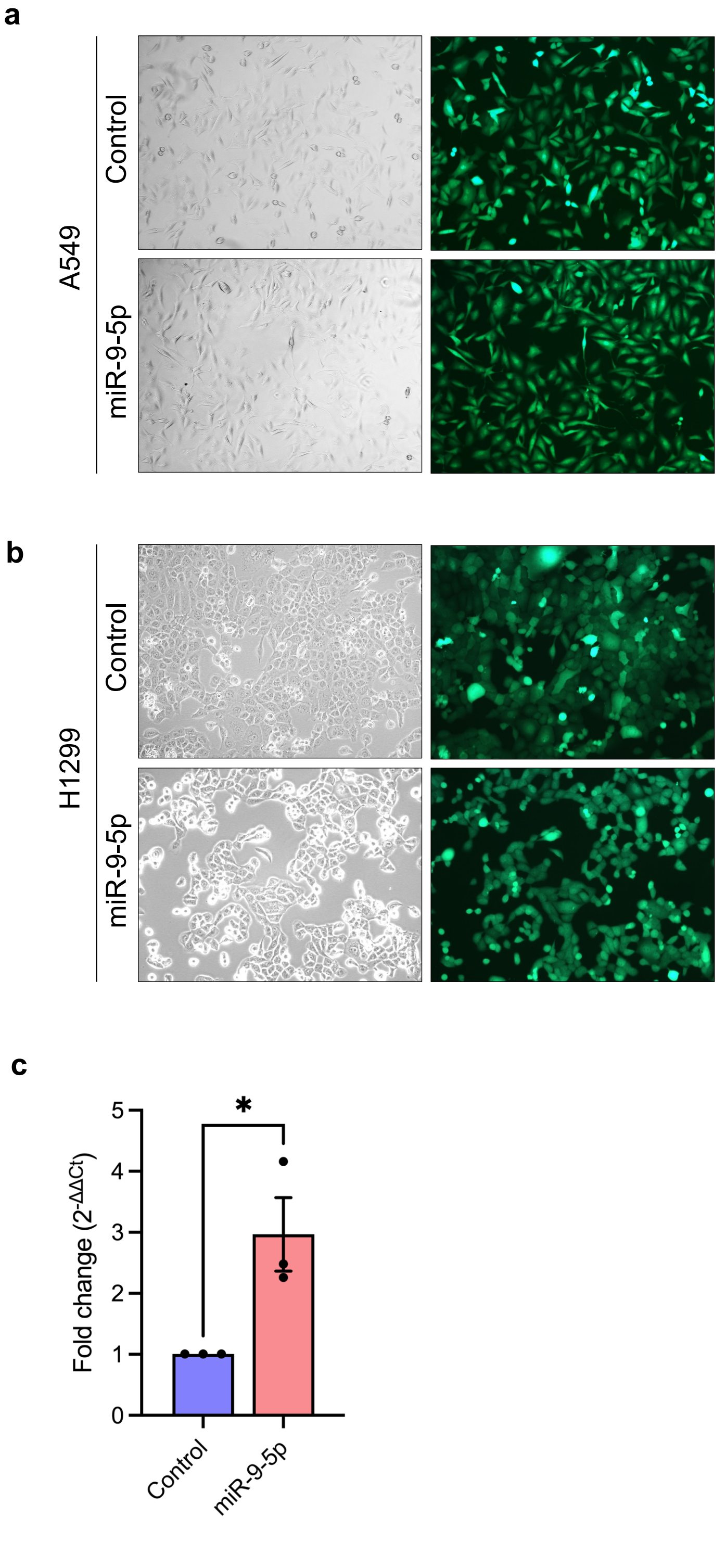

Supplementary figure 3

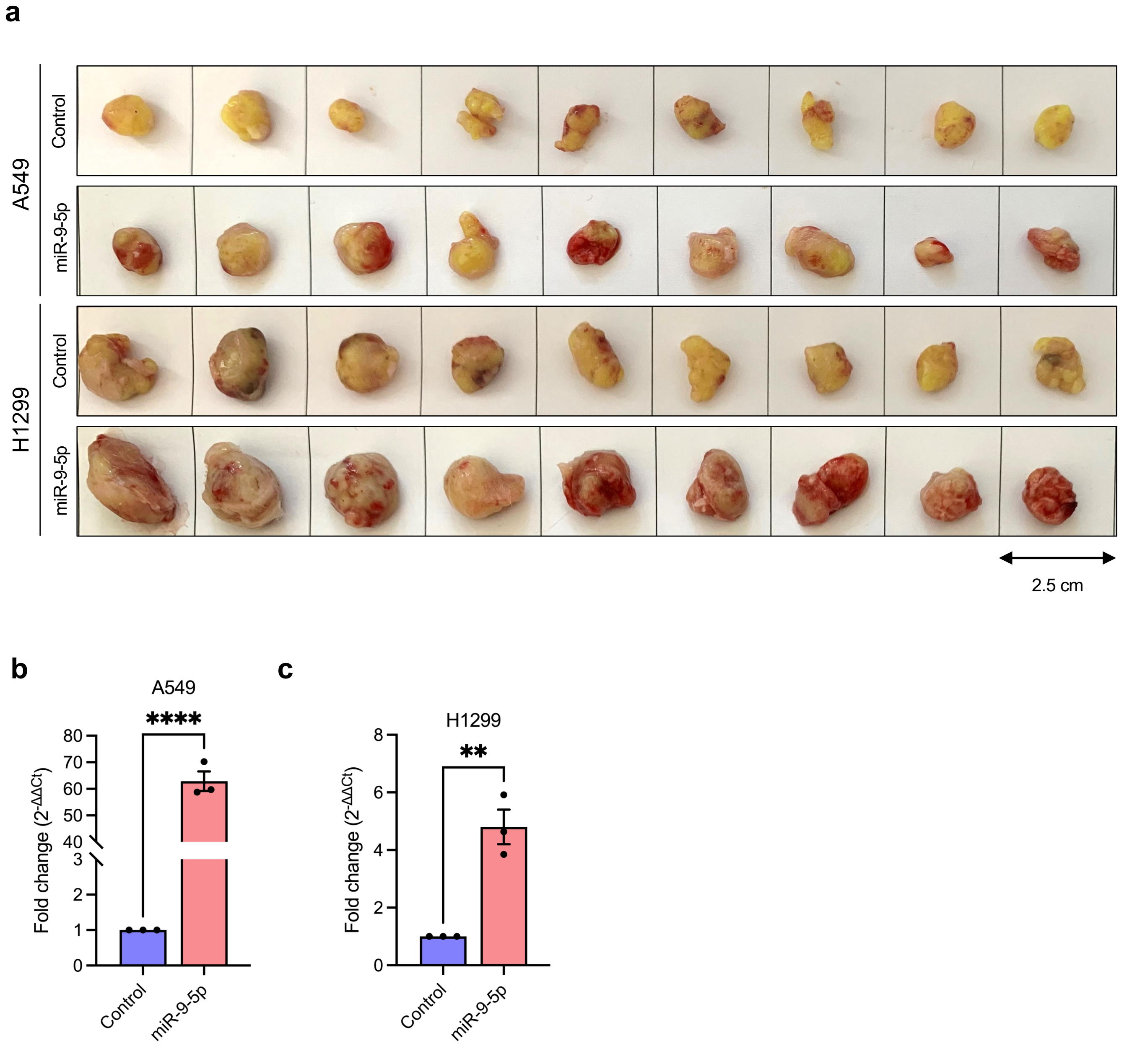

Supplementary figure 4

a. Upregulated genes in A549 miR-9-5p overexpressing samples

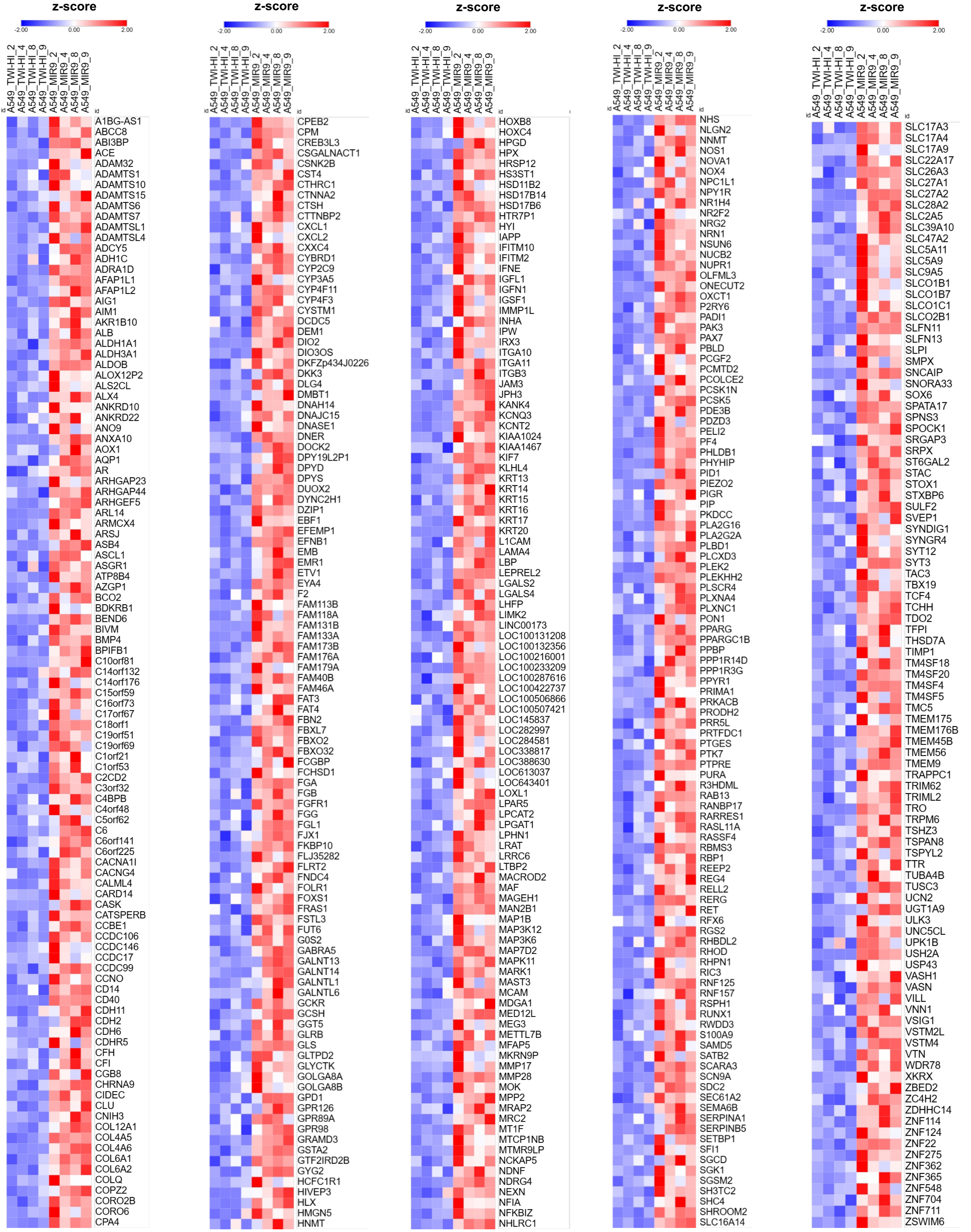

Supplementary figure 4

b. Downregulated genes in A549 miR-9-5p overexpressing samples

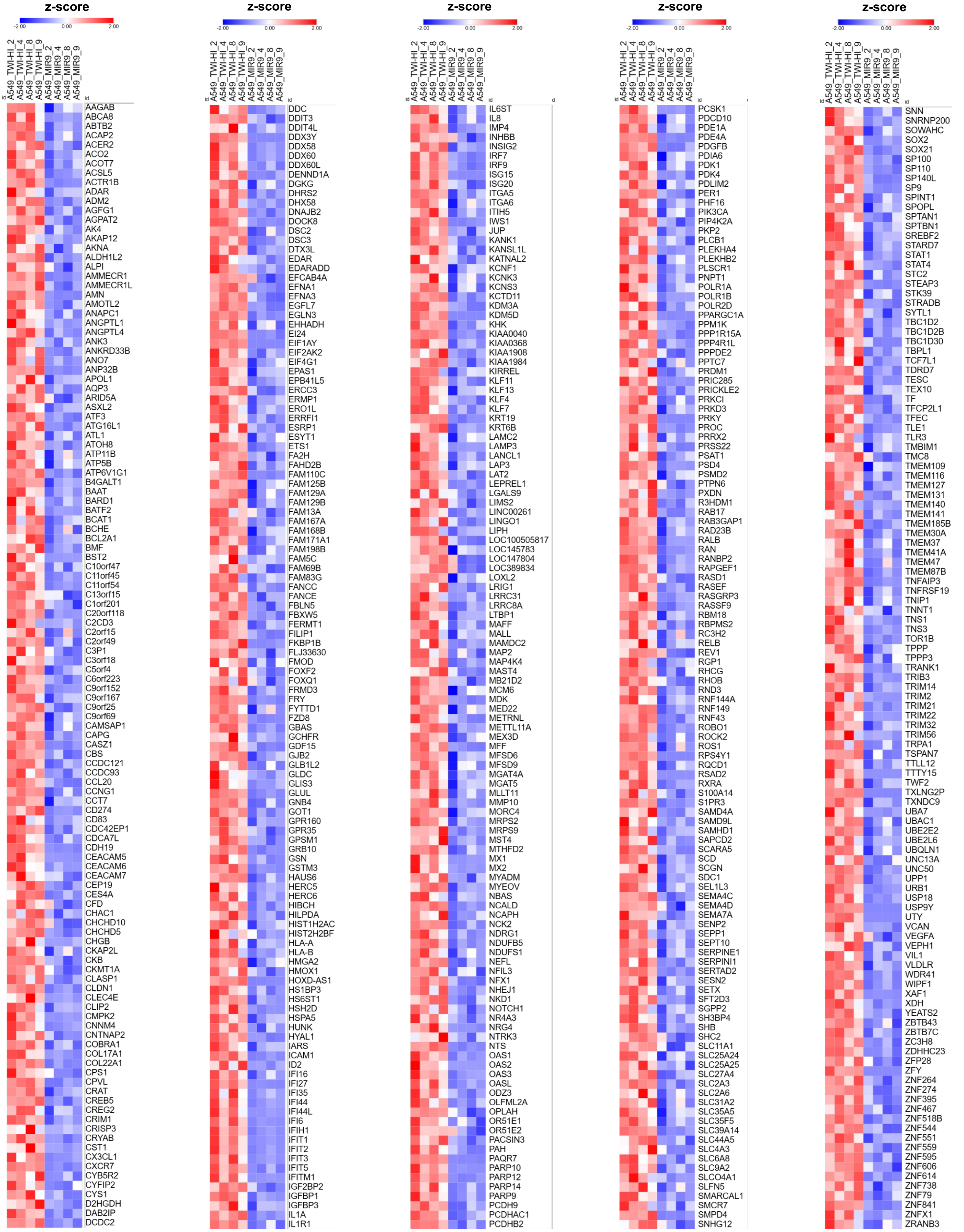

Supplementary figure 4

c. Upregulated genes in H1299 miR-9-5p overexpressing samples

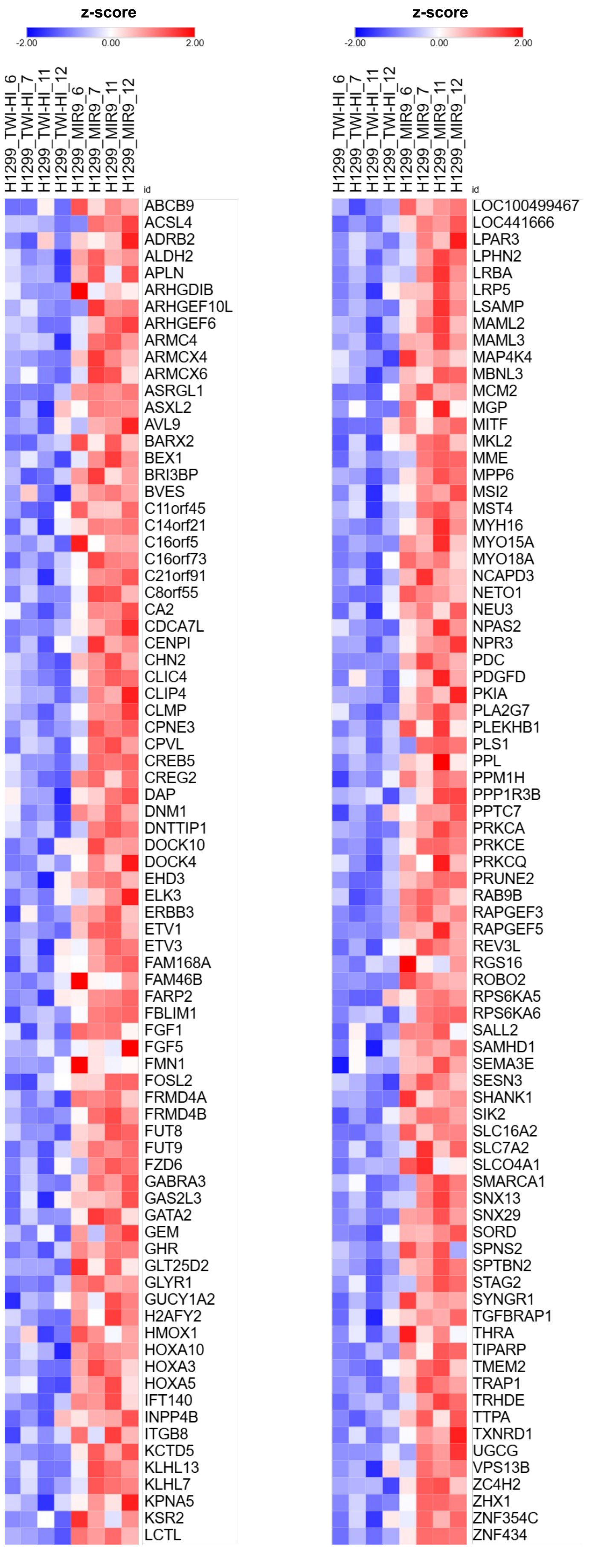

Supplementary figure 4

d. Downregulated genes in H1299 miR-9-5p overexpressing samples

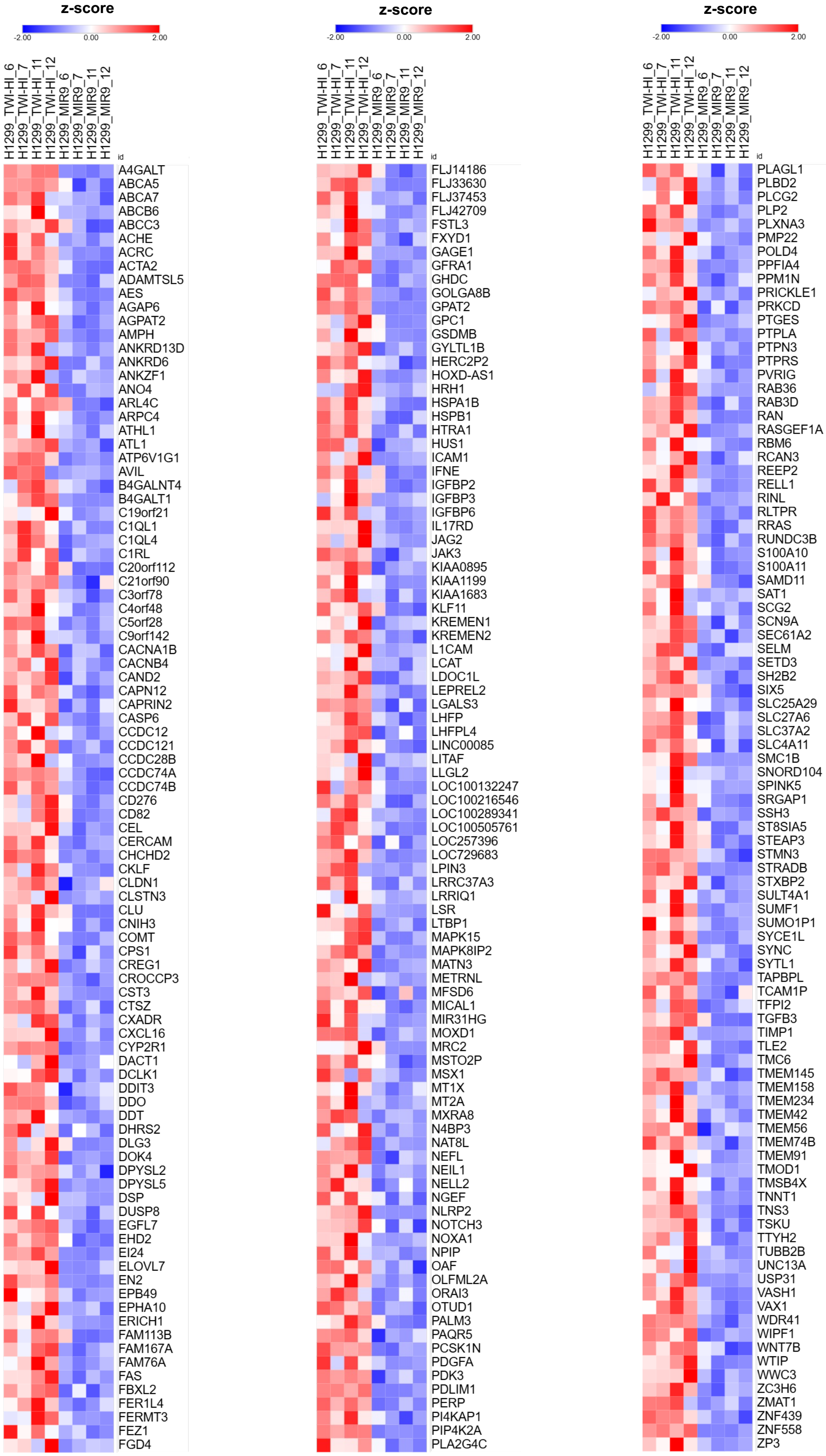

Supplementary figure 4

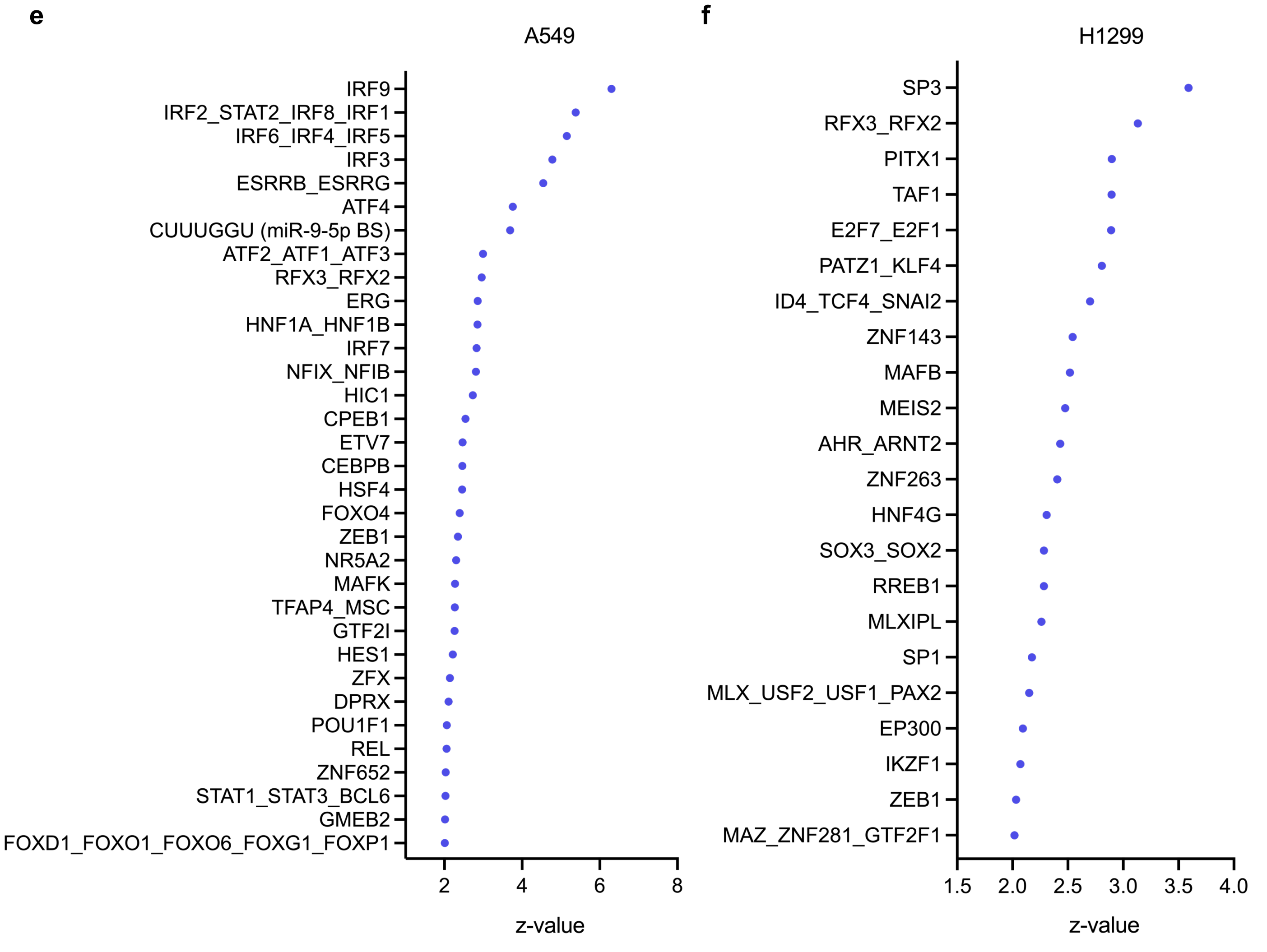

Supplement: Supplementary file 1 [file 41420_2026_3195_MOESM1_ESM.pdf]
