# Supplementary material for: The IKKα-regulated microRNA miR-9-5p mediates lung cancer growth and invasiveness via CDH1/Wnt/β-catenin signalling
Source: Cell Death Discov. 2026 Jun 17;12:346. doi: 10.1038/s41420-026-03195-8 (PMC13507200; doi:10.1038/s41420-026-03195-8)
Supplement: Supplementary file 2 [file 41420_2026_3195_MOESM2_ESM.pdf]

Supplementary Information

Supplementary Methods

Nanostring miRNA profiling

Normalisation of the reads was performed in the raw data before threshold application, using as a background cut-off the mean of negative probe signals (exploratory study). RNAs following hybridisation reactions were processed using the nCounter Prep Station and subsequently the nCounter Digital Analyser. Levels of miRNAs (n = 800) were analysed using the nSolver software, v1.1 (Nanostring Technologies Inc, Seattle, WA, USA). Normalisation was performed using all miRNAs with coefficient of variation less than 50%.

RNA sequencing

RNASeq library preparation was carried out with the NEBNext Ultra II directional library prep kit for Illumina, library quality control was performed with the qubit HS spectrophotometric method and the Agilent Bioanalyzer DNA1000 chip. Next generation sequencing (NGS) was performed using the Illumina NextSeq 2000 sequencer. Quality was assessed with FastQC. Bioinformatics analyses were performed from within the Galaxy suite. To identify differentially expressed genes, sequencing reads were mapped to the human genome (hg19) using HISAT2 with the reverse strand option. HTseq-count was used in reverse mode for counting reads falling inside genes. Differential gene expression analysis was performed using DESeq2 and an adjusted p-value of 0.05 was used as a threshold for significance.

Antibodies used for western blotting

The antibodies used were the following: mouse IKKα (Novus Biologicals #14A231) at 1:1000; rabbit IKKβ (Cell Signaling Technology (CST) #2678T) at 1:1000; rabbit phospho-IKKα/β (Ser176/180) (CST #2697) at 1:1000; mouse IκBα (CST #4814) at 1:1000; rabbit phospho-IκBα (Ser32) (CST #2859) at 1:1000; rabbit p100/p52 (NFKB2) (ProteinTech #15503-1-AP) at 1:1000; mouse Vinculin (SantaCruz Biotechnology #sc-73614) at 1:500; mouse E-Cadherin

(CST #3195) at 1:500; mouse N-Cadherin (Santa Cruz Biotechnology #sc-59987) at 1:500; rabbit Vimentin (CST #5741) at 1:1000; rabbit non-phospho-β-catenin (non-p-S45) (CST #19807) at 1:1000; rabbit β-catenin (ProteinTech #51067-2-AP) at 1:20000; rabbit phospho-GSK-3β (S9) (CST #9323) at 1:2000; rabbit GSK-3β (CST #9315) at 1:1000; rabbit phospho-Akt (S473) (CST #9271) at 1:500; rabbit Akt1 (CST #2938) at 1:1000; mouse GAPDH (Santa Cruz Biotechnology #sc-47724) at 1:3000; and mouse β-actin (Sigma #A5441) at 1:1000. The following secondary antibodies were used: horse horseradish peroxidase (HRP)-conjugated anti-mouse IgG (CST #7076) and goat HRP-conjugated anti-rabbit IgG (CST #7074).

Primers used for RT-qPCR

For the miRNA RT-qPCR reactions, all the primers were designed and made by Qiagen: hsa-miR-9-5p (#YP00204513), hsa-miR-222-3p (#YP00204551) and 5S rRNA (hsa) (V00589). For the miRNA RT-qPCRs, mir-222-3p and 5S rRNA were used as internal controls. The following primers were used for the RT-qPCR reactions:

CLDN1

Forward: 5’ CTGTCATTGGGGGTGCGATA 3’

Reverse: 5’ CTGACCAAATTCGTACCTGGC 3’

IGFBP3

Forward: 5’ TAAGAAAAAGCAGTGTCGCCC 3’

Reverse: 5’ AGGCGTCTACTTGCTCTGC 3’

LTBP1

Forward: 5’ CGGCTGAGTCAAACGAACAAA 3’

Reverse: 5’ GAATCCTTCAGGGGGCAGAG 3’

GAPDH

Forward: 5’ CAGCAAGAGCACAAGAGGAAG 3’

Reverse: 5’ TGGTACATGACAAGGTGCGG 3’

Supplementary Figure Legends

Supplementary Fig. 1. (**a**) Western blot for the expression of IKKβ in A549 control and $IKK\beta^{KD}$ cell lines. Actin was used as a loading control. (b) Graphical representation of network between differentially expressed miRNAs in A549 $IKK\alpha^{KD}$ cells and their target genes. Analysis was performed with miRNet, using experimentally supported mRNA targets by TarBase v9.0. (**c**) KEGG pathway analysis of common targets of the differentially expressed miRNAs in A549 $IKK\alpha^{KD}$ cells. Analysis was performed by ShinyGO. FDR-adjusted p-values are shown. (**d**) Western blot of phopsho-IKKα/β (Ser176/180) and phospho-IκBα (Ser32) in A549 cells treated with 10 ng/ml TNFα and 2 μM Sulfasalazine. Vinculin is used as a loading control.

Supplementary Fig. 2. (**a, b**) Images of (**a**) A549 and (**b**) H1299 clonal cell lines expressing the control empty vector and miR-9-5p in addition to EGFP. (**c**) RT-qPCR analysis of miR-9-5p expression in H1299 control and miR-9-5p overexpressing cell line.

Supplementary Fig. 3. (**a**) Images of A549 and H1299 control and miR-9-5p overexpressing tumours after surgical excision. (**b, c**) RT-qPCR of tumour RNA showing miR-9-5p upregulation persisted to the end of the experiment in (**b**) A549 and (**c**) H1299 samples. Graphs show mean +/- SEM (two-tailed unpaired Student's t test, **p<0.01, ****p<0.0001, n = 3 biological repeats).

Supplementary Fig. 4. (**a, b, c, d**) Heatmaps of differentially expressed genes in (**a, b**) A549 and (**c, d**) H1299 miR-9-5p overexpressing tumours. Analysis was performed by DESeq2. (**e, f**) Transcription factor motif enrichment analysis with ISMARA of differentially expressed genes in (**e**) A549 and (**f**) H1299 miR-9-5p tumours (46). Significantly enriched (z-value > 2) TF motifs are presented in the graphs.

Supplementary Table Legends

Table S1

Table showing normalised reads of A549 control, IKKα knockdown or IKKβ knockdown cells from Nanostring miRNA profiling. Table also shows fold change normalised to control samples.

Table S2

RNA sequencing analysis with DESeq2 of xenografts generated with control and miR-9-5p overexpressing A549 cells.

Table S3

RNA sequencing analysis with DESeq2 of xenografts generated with control and miR-9-5p overexpressing H1299 cells.
