# Supplementary material for: The IKKα-regulated microRNA miR-9-5p mediates lung cancer growth and invasiveness via CDH1/Wnt/β-catenin signalling
Source: Cell Death Discov. 2026 Jun 17;12:346. doi: 10.1038/s41420-026-03195-8 (PMC13507200; doi:10.1038/s41420-026-03195-8)

Figure 1a

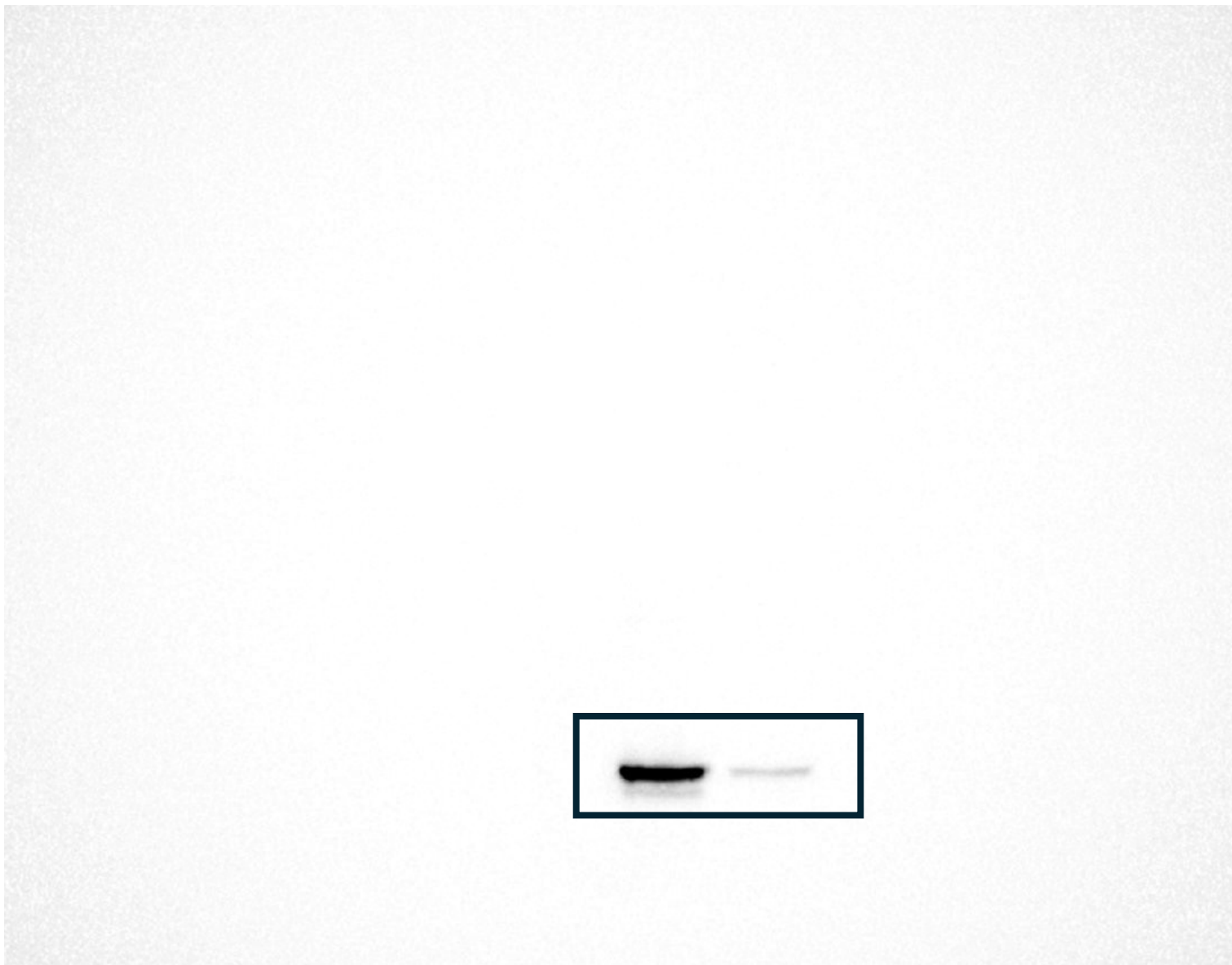

IKKα

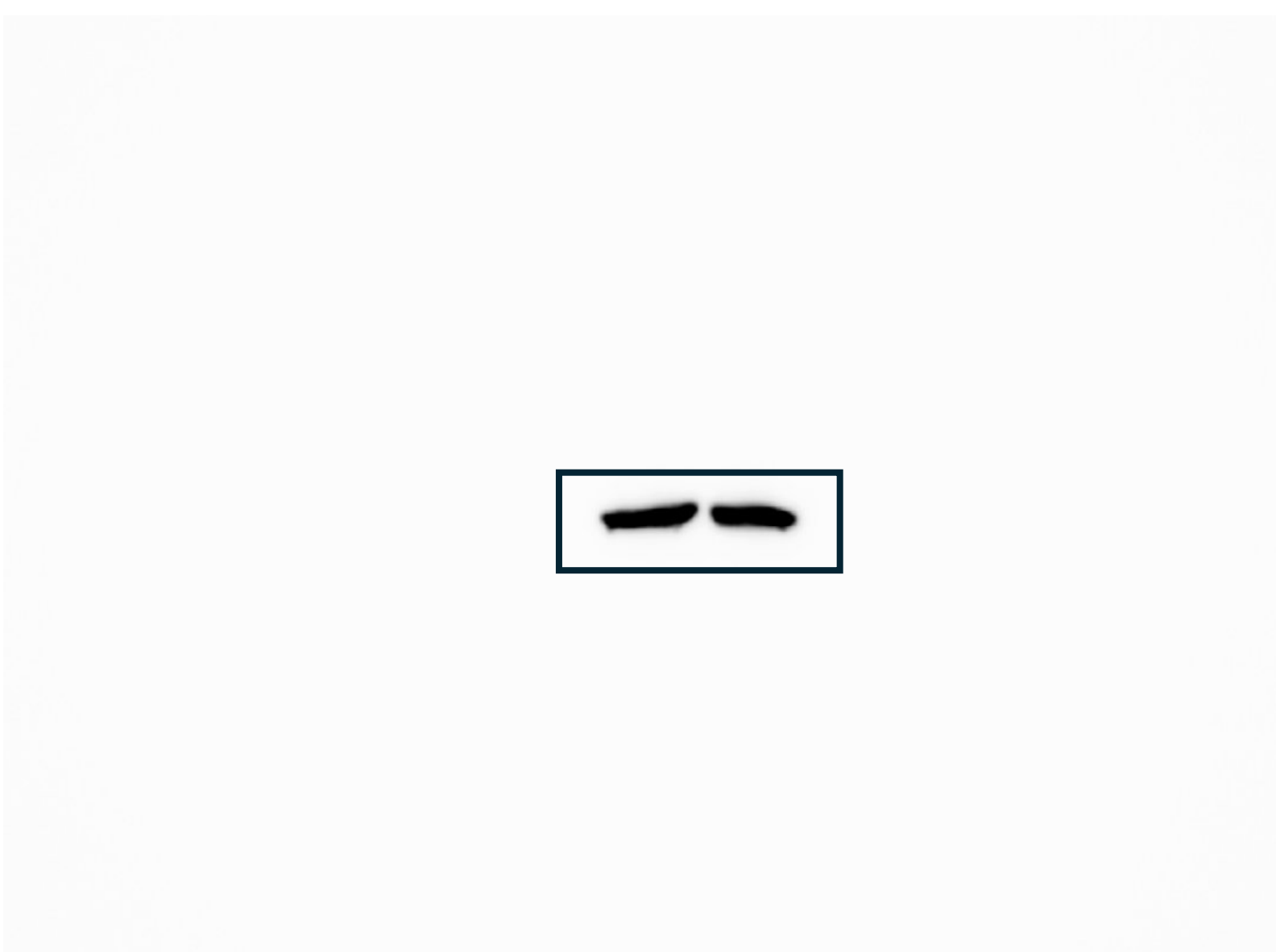

β-actin

Figure 1d

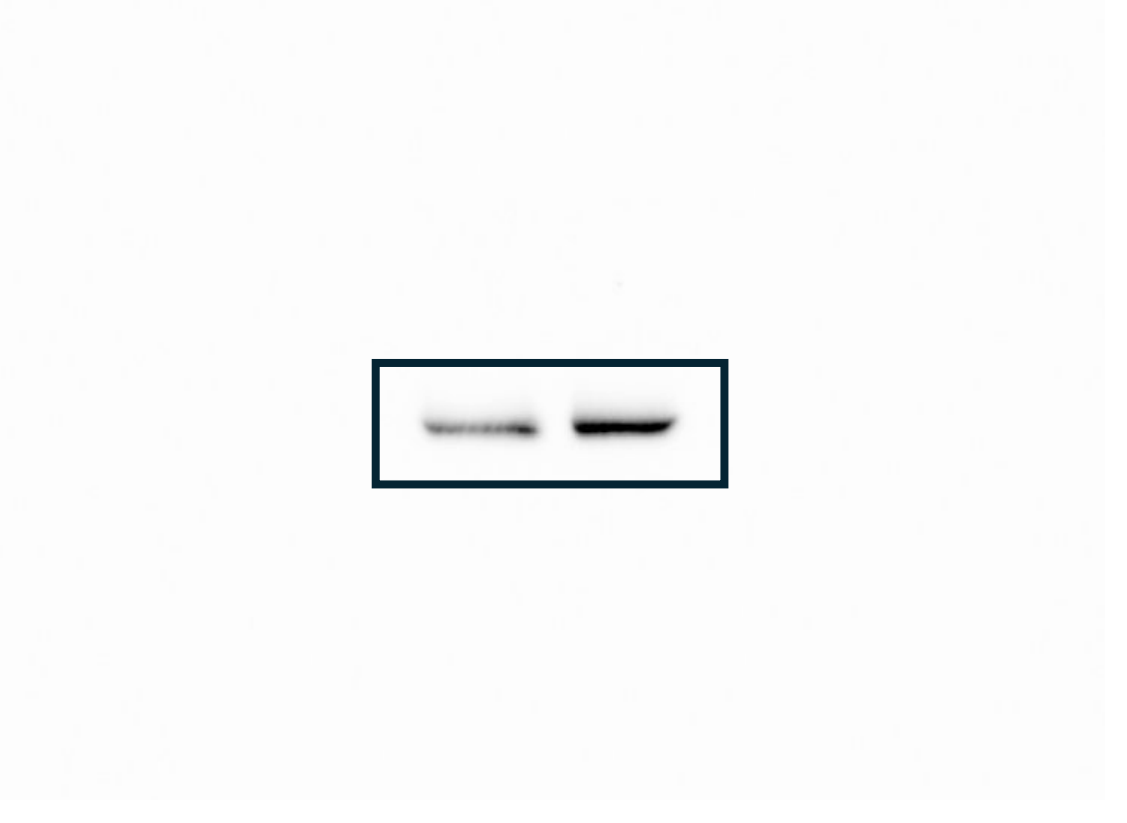

IKKβ

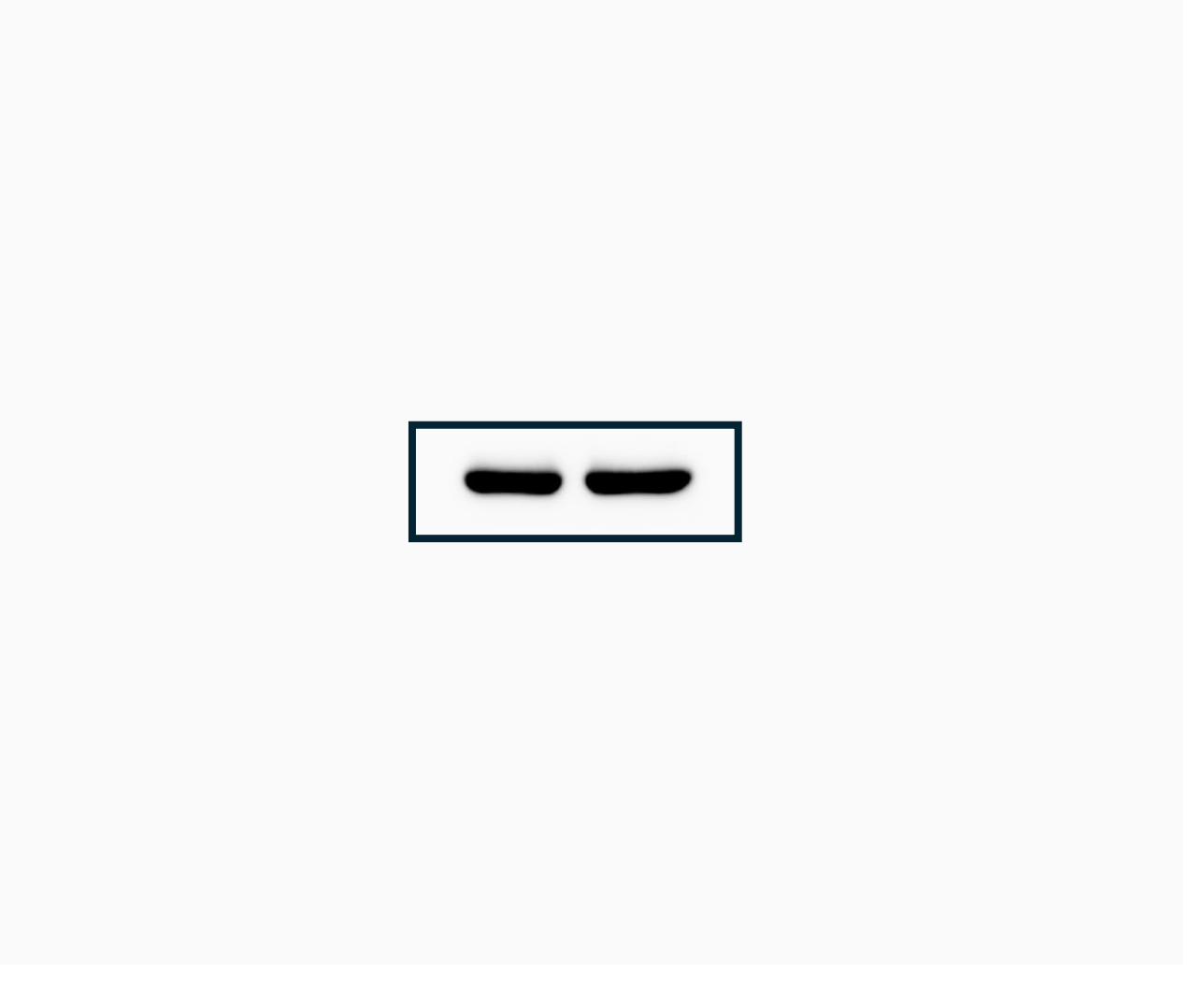

β-actin

Figure 1g

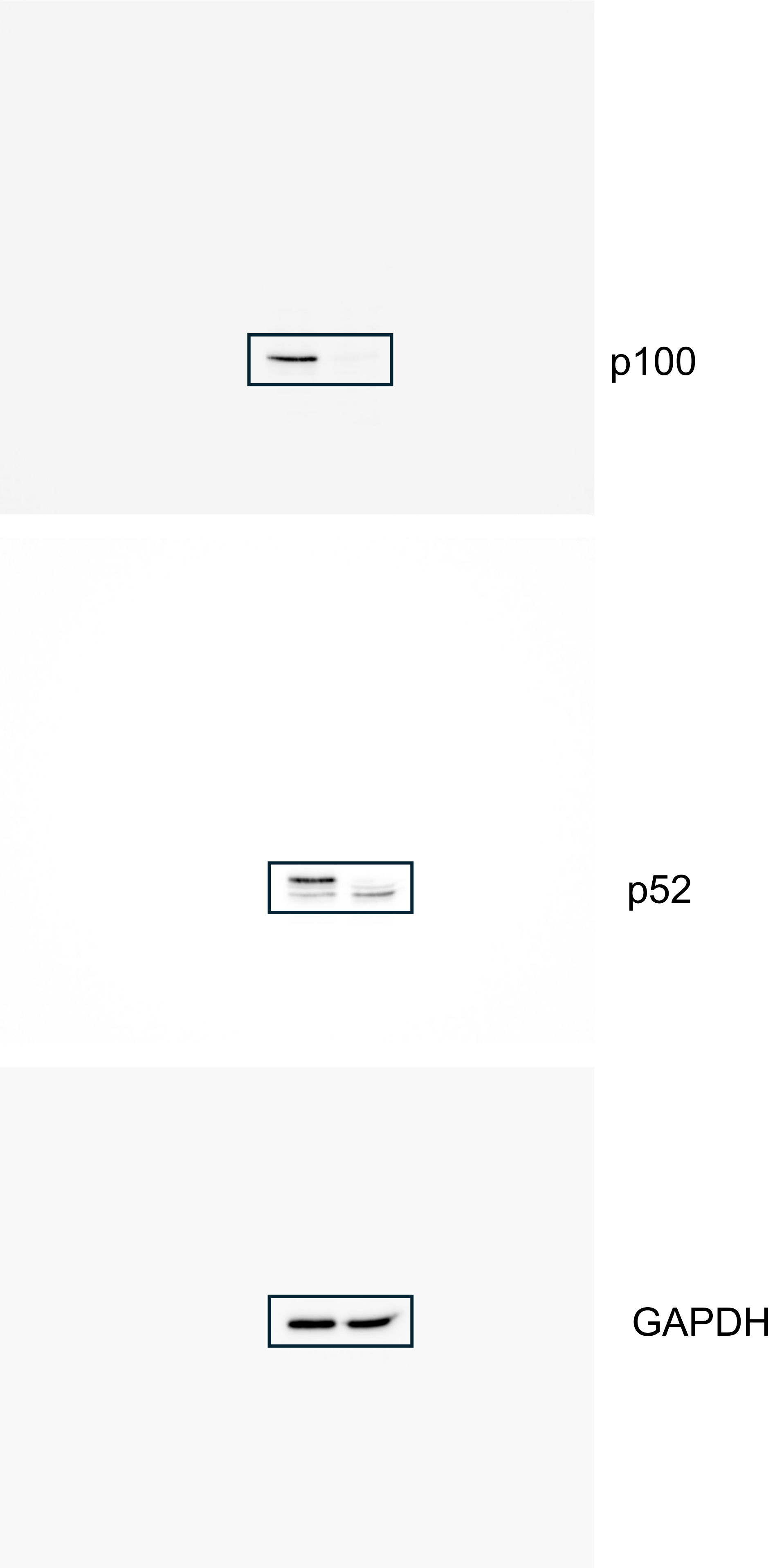

Figure 1i

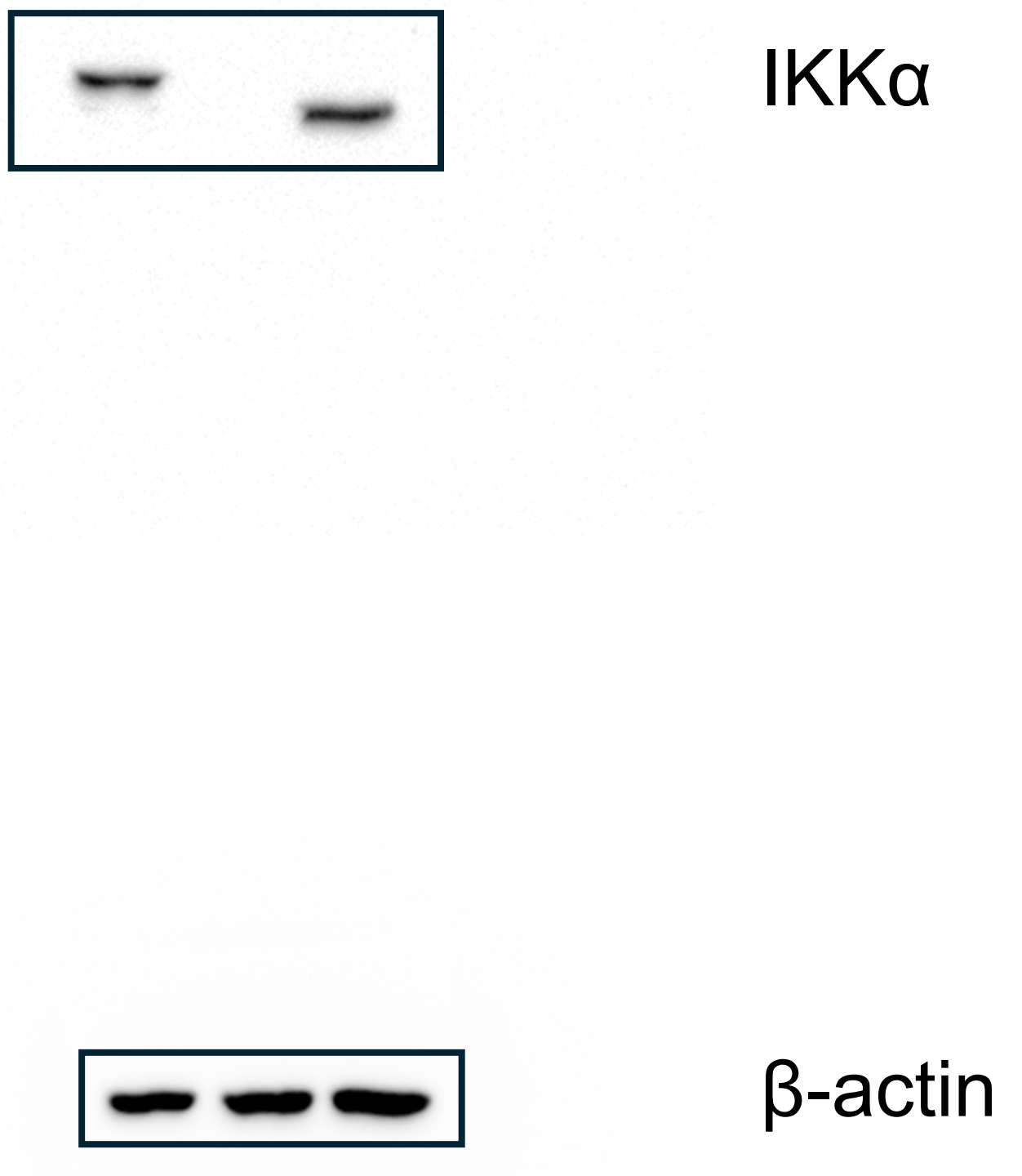

Figure 2f

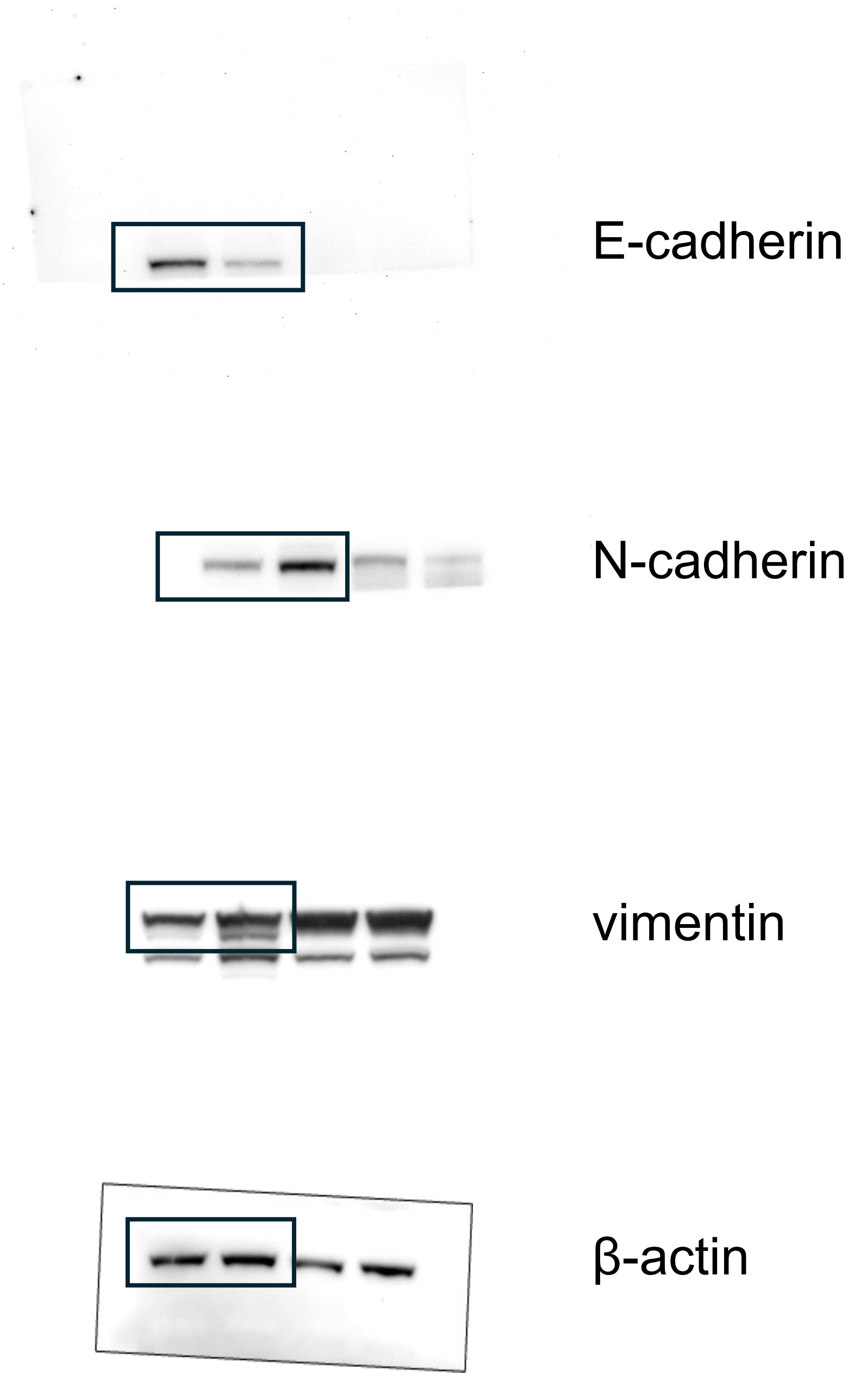

Figure 2g

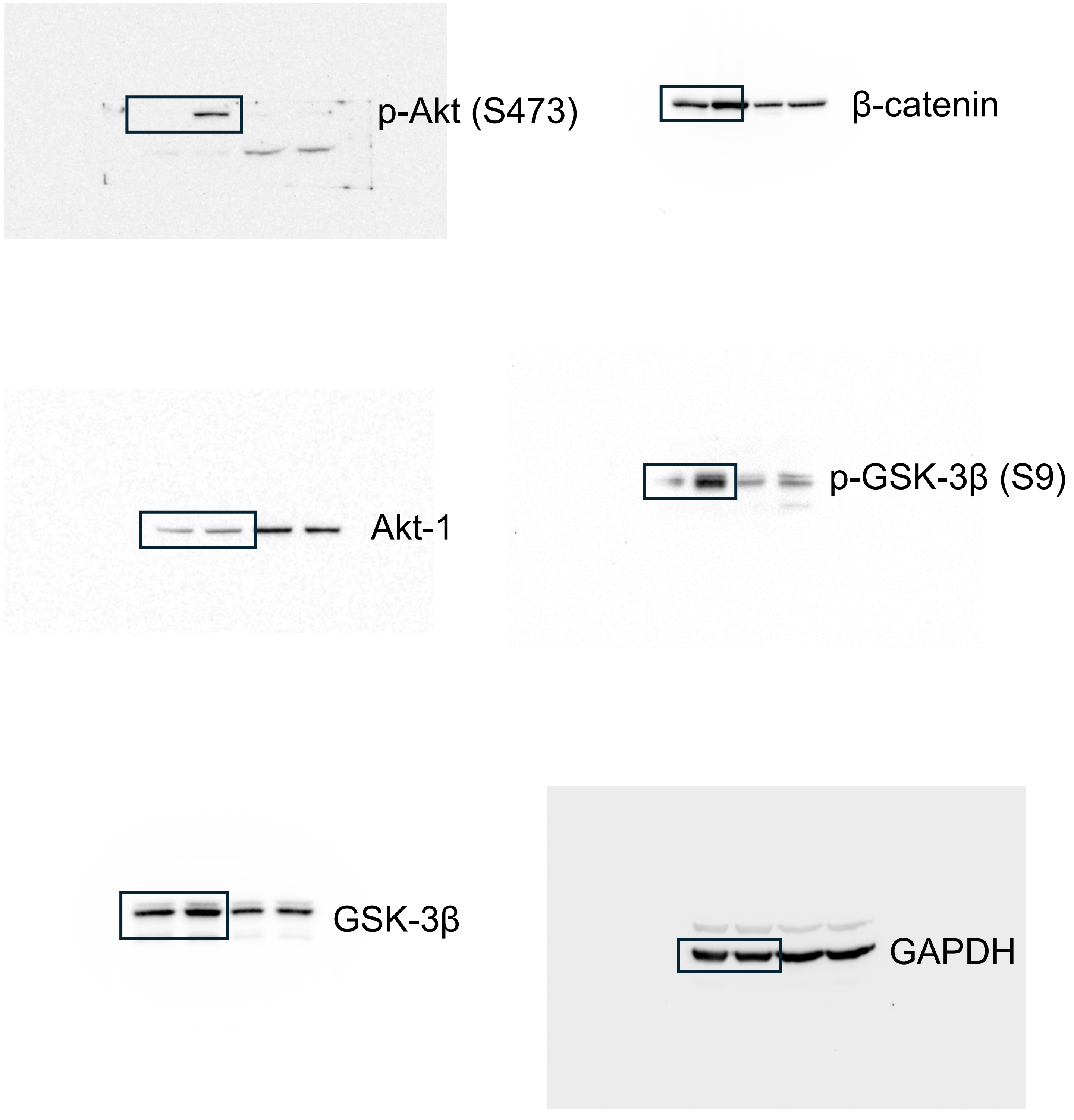

Figure 2g continued
*Non-p-S45 β catenin was run on a separate gel using the same samples and a loading control as it overlaps in weight with total β catenin

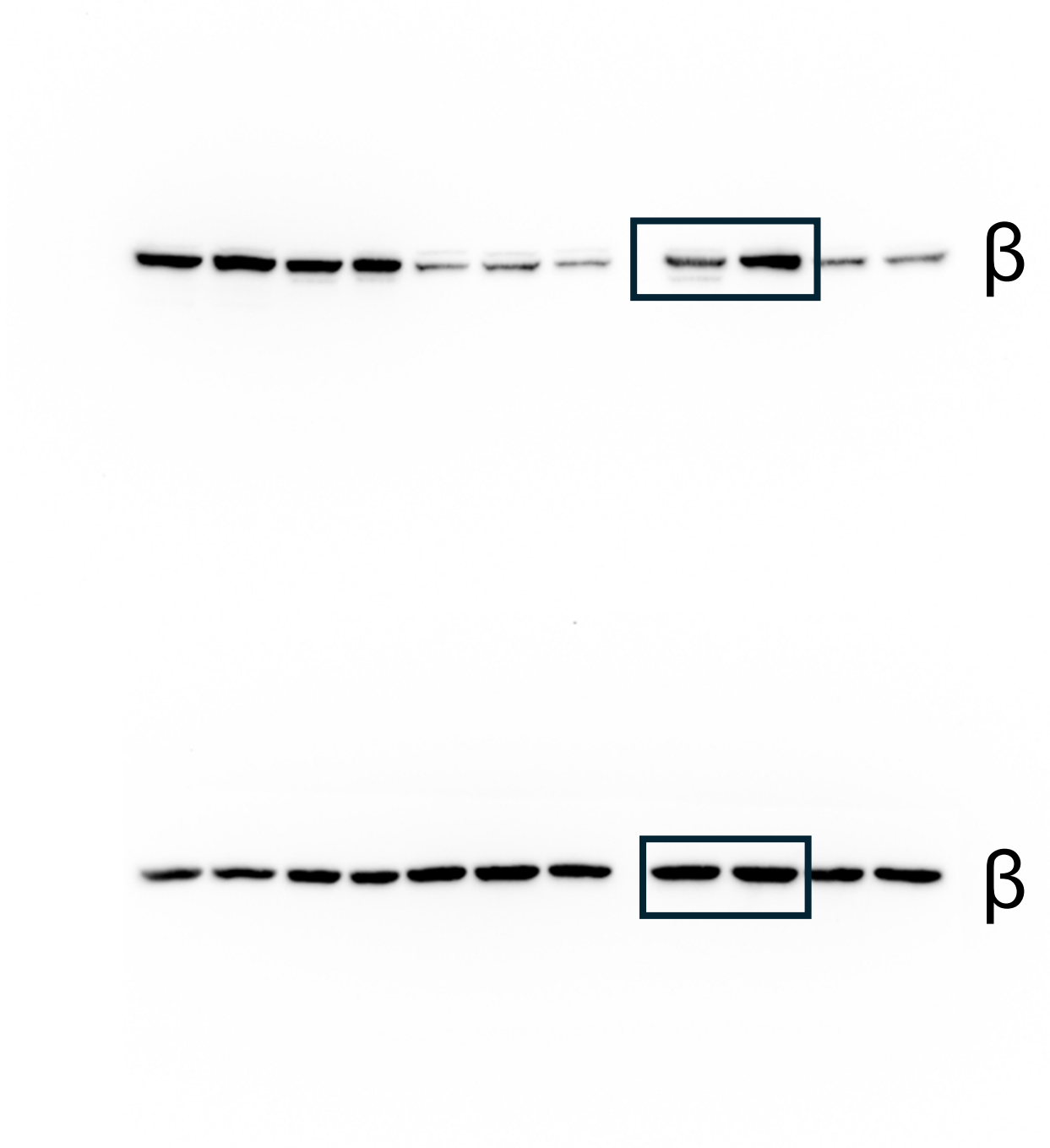

β catenin (non p-S45)

β actin (not shown in figure)

Supplementary Figure 1a

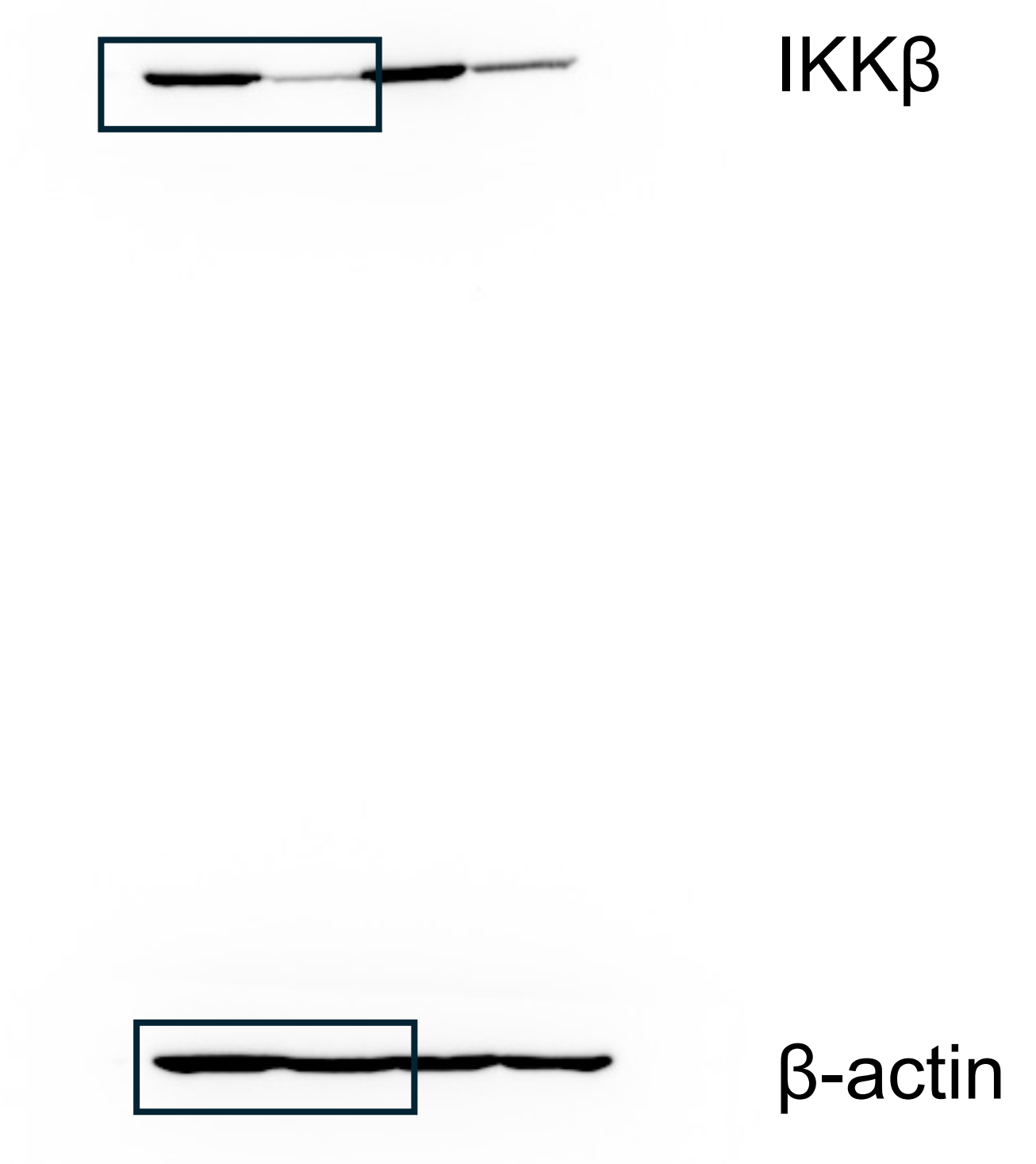

Supplementary Figure 1d

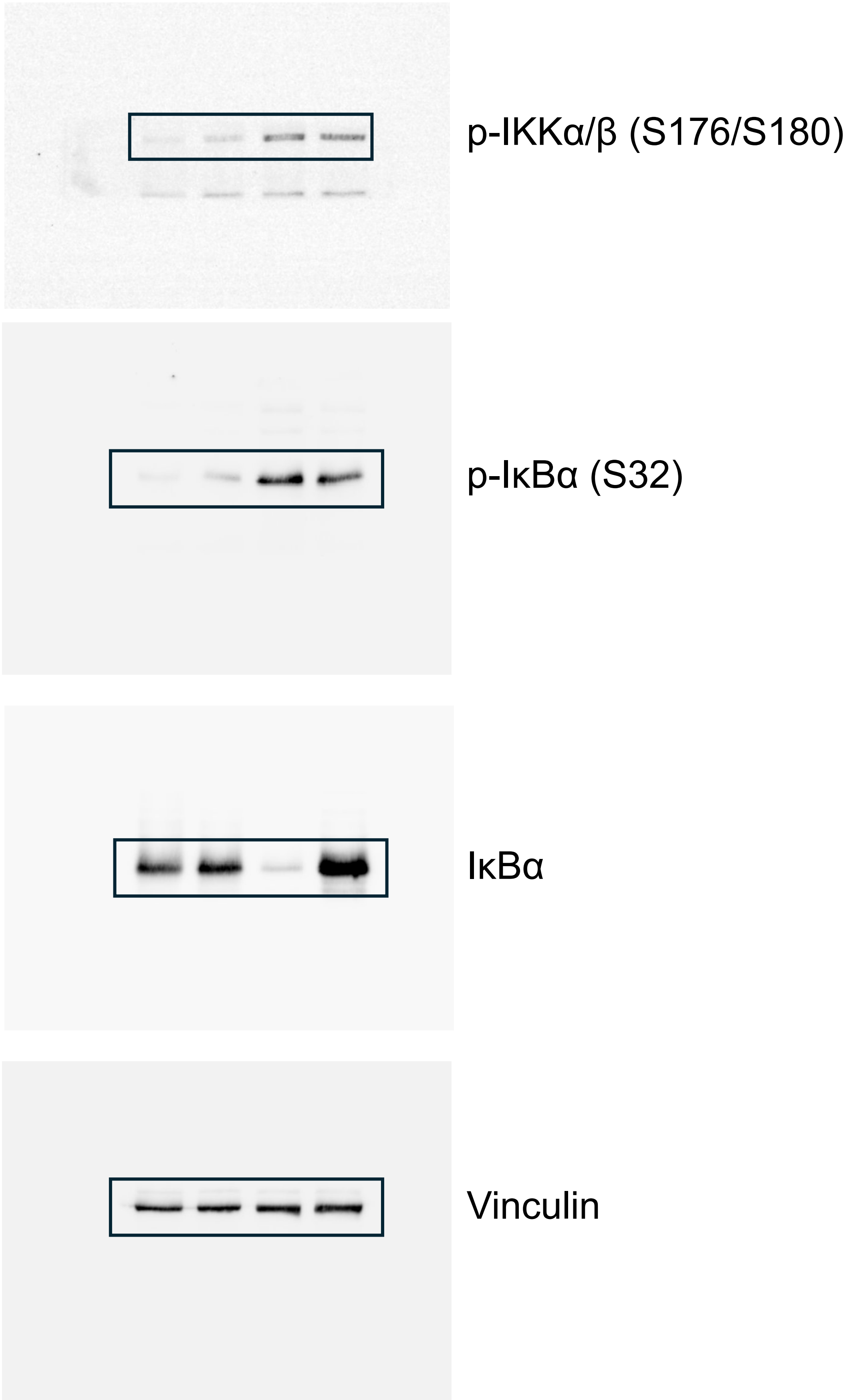

Supplement: Supplementary file 3 [file 41420_2026_3195_MOESM6_ESM.pdf]
